# The osteogenic differentiation of human adipose-derived stem cells is regulated through the let-7i-3p/LEF1/β-catenin axis under cyclic strain

**DOI:** 10.1186/s13287-019-1470-z

**Published:** 2019-11-21

**Authors:** Yadong Luo, Ran Ge, Heming Wu, Xu Ding, Haiyang Song, Huan Ji, Meng Li, Yunan Ma, Sheng Li, Chenxing Wang, Hongming Du

**Affiliations:** 1 Department of Oral and Maxillofacial Surgery, Affiliated Stomatological Hospital of Nanjing Medical University, Hanzhong Road No.136, Nanjing, 210029 Jiangsu Province People’s Republic of China; 2 Oral Disease Key Laboratory of Jiangsu Province, Nanjing Medical University, Nanjing, 210029 Jiangsu Province People’s Republic of China; 3 Department of Nuclear Medicine, Fujian Medical University Union Hospital, Fuzhou, 350001 Fujian Province People’s Republic of China

**Keywords:** hASC osteogenic differentiation, Cyclic strain, Wnt/β-catenin pathway, Let-7i-3p, LEF1

## Abstract

**Background:**

The Wnt/β-catenin pathway is involved in the osteogenic differentiation of human adipose-derived stem cells (hASCs) under cyclic strain. Very little is known about the role of microRNAs in these events.

**Methods:**

Cells were obtained using enzyme digestion methods, and proliferation was detected using Cell Counting Kit 8. Cell cycles and immunophenotypes were detected by flow cytometry. The multilineage potential of hASCs was induced by induction media. Cyclic strain was applied to hASCs (0.5 Hz, 2 h/day, 6 days) to induce osteogenic differentiation and miRNA changes. Bioinformatic and dual-luciferase analyses confirmed lymphoid enhancer factor 1 (LEF1) as a potential target of let-7i-3p. The effect of let-7i-3p on LEF1 in hASCs transfected with a let-7i-3p mimic and inhibitor was analyzed by immunofluorescence. hASCs were transfected with a let-7i-3p mimic, inhibitor, or small interfering RNA (siRNA) against LEF1 and β-catenin. Quantitative real-time PCR (qPCR) and western blotting were performed to examine the osteogenic markers and Wnt/β-catenin pathway at the mRNA and protein levels, respectively. Immunofluorescence and western blotting were performed to confirm the activation of the Wnt/β-catenin pathway.

**Results:**

Flow cytometry showed that 82.12% ± 5.83% of the cells were in G1 phase and 17.88% ± 2.59% of the cells were in S/G2 phase; hASCs were positive for CD29, CD90, and CD105. hASCs could have the potential for osteogenic, chondrogenic, and adipogenic differentiation. MicroRNA screening via microarray showed that let-7i-3p expression was decreased under cyclic strain. Bioinformatic and dual-luciferase analyses confirmed that LEF1 in the Wnt/β-catenin pathway was the target of let-7i-3p. Under cyclic strain, the osteogenic differentiation of hASCs was promoted by overexpression of LEF1and β-catenin and inhibited by overexpression of let-7i-3p. hASCs were transfected with let-7i-3p mimics and inhibitor. Gain- or loss-of-function analyses of let-7i-3p showed that the osteogenic differentiation of hASCs was promoted by decreased let-7i-3p expression and inhibited by increased let-7i-3p expression. Furthermore, high LEF1 expression inactivated the Wnt/β-catenin pathway in let-7i-3p-enhanced hASCs. In contrast, let-7i-3p inhibition activated the Wnt/β-catenin pathway.

**Conclusions:**

Let-7i-3p, acting as a negative regulator of the Wnt/β-catenin pathway by targeting LEF1, inhibits the osteogenic differentiation of hASCs under cyclic strain in vitro.

## Introduction

Bone tissue engineering can be used to repair bone defects induced by various factors by construction of functional bone tissue in vitro [1]. Seed cells, bioscaffold materials, and cell and bioscaffold materials in combination are the three significant components of bone tissue engineering [2]. Among them, research on seed cells is one focus of the bone tissue engineering field [3].

Bone marrow mesenchymal stem cells (BMSCs) are currently the most widely used seed cells in the field of bone tissue engineering [4]. However, there are still some problems in the practical application of BMSCs, such as limited cell acquisition, complex cellular components, and decreased osteogenic differentiation in aging cell donors [5]. In light of this, researchers began looking for other types of adult stem cells as seed cells for bone tissue engineering. In 2001, Zuk et al. first obtained human adipose-derived stem cells (hASCs), which have multilineage differentiation potential, including osteogenic, chondrogenic, and adipogenic differentiation abilities [6]. The advantages of ASCs over BMSCs include abundant cell sources, easy access, good expansion, and stable osteogenic differentiation activity in vitro [7, 8]. While numerous papers also describe the disadvantages of ASC vs BMSC osteogenesis [9], many scholars still believe that ASCs are a valuable new seed cell source in bone tissue engineering [10, 11].

How to promote the osteogenic differentiation of ASCs in vitro has become an important research area for bone tissue engineering. Mechanical loading plays an essential role in cellular biological responses, such as the regulation of protein synthesis, cell proliferation, and differentiation [12]. Some studies have shown that tensile stress can promote the osteogenic differentiation of adult MSCs, including hASCs [13]. The above results suggest that tensile stress stimulation can promote the osteogenic differentiation of ASCs.

The Wnt signaling pathway is involved in the regulation of many critical biological processes, including embryogenesis and development, stem cell maintenance and differentiation, energy metabolism balance, and tissue stability maintenance. The Wnt/β-catenin pathway plays a complicated role in bone biology. Some syndromes show low bone mass and, consequently, frequent bone fractures caused by loss of functional mutations of the coreceptor LRP5 [14], while gain-of-function mutations of the LRP5 receptor lead to high bone mass [15]. Bone thickness was shown to be decreased in mice Wnt-16 knockout mice [16]. In contrast, LEF1 was shown to be overexpressed in the initial and later phases of bone repair [17]. The activated Wnt/β-catenin pathway can promote the osteogenic differentiation of BMSCs [18]. Although the activation of the Wnt/β-catenin pathway results in bone formation, increased bone mass results from excessive bone formation rather than bone resorption in both situations [19]. Previous studies have also found that tensile stress activates the Wnt/β-catenin pathway, which promotes osteogenic differentiation [20, 21].

MicroRNAs (miRNAs) are highly conserved single-stranded noncoding RNA molecules consisting of 18–22 nucleotides. MiRNAs bind to the 3′UTRs of their target mRNAs, inhibiting their translation or promoting their degradation [22]. miRNAs are involved in complex biological processes in organisms, including cell division, differentiation, apoptosis, and organ development [22]. Notably, miRNAs are involved in regulating the osteogenic differentiation of adult MSCs by regulating the expression of Wnt/β-catenin pathway molecules. For example, miR-335-5p activates the Wnt/β-catenin pathway by inhibiting Dkk1 expression, thereby promoting osteogenic differentiation [23]. miR-138 and miR-30e regulate the expression of FAK and LRP6, respectively, thereby inhibiting the osteogenic differentiation of MSCs [24, 25]. Although existing research results indicate that miRNAs play an essential role in cell osteogenic differentiation of cells by regulating the Wnt/β-catenin pathway, research in this field is still incomplete due to the diversity of miRNAs and their target genes, and additional studies should be performed.

In this study, a gene microarray was used to screen the altered expression levels of miRNAs in hASCs during osteogenic differentiation under cyclic strain. We analyzed miRNAs associated with Wnt/β-catenin by bioinformatics methods and found that the target gene regulated by hsa-let-7i-3p is most likely lymphoid enhancer factor 1 (LEF1) in the Wnt/β-catenin pathway. We hypothesize that cyclic strain activates the Wnt/β-catenin pathway by downregulating hsa-let-7i-3p, thereby promoting the osteogenic differentiation of hASCs. This study aimed to validate this hypothesis using cell and molecular biology methods.

## Materials

### Cell culture

Fat tissue was obtained from 6 donors who underwent flap reconstruction in the Oral and Maxillofacial Surgery Department at the Affiliated Stomatological Hospital of Nanjing Medical University, and all patients signed informed consent. The procedures were approved by the ethics committee of Nanjing Medical University. Adipose tissue was isolated from flaps and then washed three times with phosphate-buffered saline (PBS) to remove red blood cells. The adipose tissue was digested with 0.1% collagenase type I (BD, USA) at 37 °C for 1 h. After tissue digestion, the tissue was centrifuged at 300*g* for 5 min, after which the supernatant was discarded, and the pellet was resuspended twice. Resuspend cells were cultured in alpha-modified Eagle’s medium (α-MEM, Gibco BRL, USA) containing 10% fetal bovine serum (FBS; Gibco BRL, USA) at 37 °C in a 5% CO_2_ incubator. In this study, fourth-passage cells were used in the following experiments.

### Cell proliferation

Cell proliferation was detected following the manufacturer’s manual by Cell Counting Kit-8 assays (CCK-8, Beyotime, China) according to the manufacturer’s instructions. Cells were seeded into 96-well plates at 3000 cells per well and then cultured in α-MEM supplemented with 10% FBS at 37 °C in a 5% CO_2_ incubator. Cells in 6 wells were measured by adding 10 μl of Cell Counting Kit-8 solution to each well, followed by incubation at 37 °C for 2 h. Absorbance was measured by spectrophotometry (SPECTRA MAX190, Sunnyvale, CA, USA) at 450 nm. Cell counting was performed for 8 consecutive days. The growth curve of the cells was drawn according to the above results.

### Cell cycle and immunophenotype detection by flow cytometry

Cells were collected by centrifugation and fixed with precooled 70% ethanol overnight. Cells were added to 50 μL of RNase stock solution (100 μg/mL), stained with 500 μL of PBS containing 50 μg/mL ethidium bromide (PI), and incubated at 4 °C for 30 min in the dark. Finally, the fluorescence intensity of DNA-PI was detected by a FACSVerse flow cytometer (BD, USA) for analysis of cell cycle changes.

Immunophenotyping of the hASCs was performed by staining for 30 min on ice in the dark with the fluorochrome-labeled monoclonal antibodies CD29-APC (BD, USA), CD34-PE (BD, USA), CD45-PE (BD, USA), CD90-FITC (BD, USA), and CD105-PerCP cy5.5 (BD, USA). Untreated cells served as the negative control to adjust for the compensation of fluorochrome overlap. The fluorescence intensity was examined by a FACSVerse flow cytometer (BD, USA), and flow cytometry analysis was performed with FlowJo software 7.6.1 (Leonard Herzenberg, USA).

### Multilineage potential of hASCs

hASCs were induced toward adipogenic, osteogenic, and chondrogenic differentiation to confirm their multilineage potential. Adipogenic medium (10% FBS, 1 μM dexamethasone, 200 μM indomethacin, 10 mg/L insulin, and 0.5 mM 3-isobutyl-1-methylxanthine in α-MEM) was used to induce adipogenic differentiation of hASCs; after 14 days, hASCs were stained with Oil Red O to determine the formation of lipid droplets. Osteogenic medium (10% FBS, 0.1 μM dexamethasone, 10 mM β-glycerol phosphate, and 50 μM vitamin C in α-MEM) was used to induce osteogenic differentiation of hASCs; 21 days later, hASCs were stained with Alizarin Red to confirm the existence of mineralized nodules. Chondrogenic medium (10% FBS, 10 ng/ml TGF-β1, 200 μM indomethacin, 6.25 μg/ml insulin, and 50 nM vitamin C in α-MEM) was used to induce chondrogenic differentiation of hASCs; 14 days later, hASCs were stained with Alcian Blue to verify the existence of proteoglycans.

### Cyclic strain loading on hASCs

hASCs were seeded on silicone rubber BioFlex® Culture Plates (Flexcell, USA) at a density of 1.0 × 10^5^ cells/ml and incubated in α-MEM containing 10% FBS at 37 °C in a 5% CO_2_ incubator. Twenty-four hours later, stable adherent cells in the BioFlex® Culture Plates were exposed to uniaxial cyclic strain (5%, 0.5 Hz, 2 h/day) by a Flexcell® FX-5000™ Tension System (Flexcell International Corporation, USA). The control groups were cultured in α-MEM supplemented with 10% FBS at 37 °C in a 5% CO_2_ incubator for 6 days without cyclic strain loading. On the sixth day, hASCs were collected for real-time quantitative PCR (qPCR) and western blot analyses.

### Microarray detection of miRNA changes under cyclic strain

Total RNA was isolated from hASCs by TRIzol Reagent (Invitrogen, USA) after cyclic strain for 6 days. miRNAs were isolated from total RNA using an Ambion® *mir*Vana™ miRNA Isolation Kit (Thermo Fisher Scientific, USA) according to the manufacturer’s instructions. Fluorescent labeling using the T4 RNA ligase labeling method was followed by precipitation with absolute ethanol, and the samples were then blown dry. The isolated RNA was dissolved in hybridization solution and incubated overnight at 42 °C. Then, the samples were washed with 0.2% sodium dodecyl sulfate (SDS) and 2 × SSC. After drying, the slides were scanned by the LuxScan 10K-A Dual Laser Scanner (BOAO, China).

### Bioinformatics analysis

The conservative characteristics of let-7i-3p, the 3′UTR binding site for LEF1, and the targets of let-7i-3p were analyzed by the following three databases: TargetScan (http://www.targetscan.org), miRWalk (http://mirwalk.umm.uni-heidelberg.de), and miRBase (http://www.mirbase.org).

### Artificial regulation of gene expression

The following genetic research tools were used in this study: LEF1 expression plasmid (EX-LEF1), LEF1 siRNA (siLEF1), negative control expression plasmid (EX-Ctrl), negative control siRNA (siR-Ctrl), let-7i-3p mimic, let-7i-3p inhibitor, negative control of the let-7i-3p mimic (miR-Ctrl mimic) and negative control of the let-7i-3p inhibitor (miR-Ctrl inhibitor), LEF1 3′UTR-wild type (wt-LEF1), and LEF1 3′UTR-mutant (mu-LEF1). These vectors were transfected into fourth-passage hASCs using Lipofectamine™ 2000 Transfection Reagent (Invitrogen, USA) according to the manufacturer’s instructions. The let-7i-3p mimic and inhibitor, siLEF1, miR-Ctrl mimic, miR-Ctrl inhibitor, and siR-Ctrl were synthesized by GenePharma Corporation (Shanghai, China). EX-LEF1 (pEZ-M02 vector), EX-Ctrl (pReceiver-M02 vector), wt-LEF1, and mu-LEF1 were supplied by GeneCopoeia Corporation (Guangzhou, China). ORF sequence information for EX-LEF1 and EX-Ctrl is listed in the supplemental materials. Other sequences transfected in this process are listed in Table 1.

**Table 1:** The constructed sequences used in this study

Genes	Sequence	
let-7i-3p-503-3p mimic		5′-CUGCGCAAGCUACUGCCUUGCUdTdT-3′
miR-Ctrl mimic		5′-UUGUACUACACAAAAGUACUGdTdT-3′
let-7i-3p inhibitor		5′-AGCAAGGCAGUAGCUUGCGCAGdTdT-3′
miR-Ctrl inhibitor		5′-CAGUACUUUUGUGUAGUACAAdTdT-3′
SiLEF1	Sense	5′-CCGUGAAGAGCAGGCUAAATTdTdT-3′
	Antisense	5′-UUUAGCCUGCUCUUCACGGTTdTdT-3′
siR-Ctrl	Sense	5′-UUCUCCGAACGUGUCACGUdTdT-3′
	Antisense	5′-ACGUGACACGUUCGGAGAAdTdT-3′

To change the expression of β-catenin, we transduced lentiviral particles containing an shRNA targeting β-catenin or an shRNA control (Mission Lentiviral Transduction Particles from Sigma-Aldrich, St Louis, MO, USA) into SKOV3 cells. Lentiviral-transduced SKOV3 cells were selected with puromycin (1.5 μM/mL). Transient transfection was performed using a pool of 4 short interfering RNAs (siRNAs) targeting β-catenin (Dharmacon, Pittsburgh, PA, USA; siGenome SMART pool) or individual siRNA sequences (#1: GCGUUUGGCUGAACCAUCA and #2: UAAUGAGGACCUAUACUUA, Dharmacon) and DreamFECT transfection reagent (Oz Biosciences, Marseille, France). The scrambled siRNA pool (Dharmacon) was used as a control.

### Dual-luciferase reporter assay

Sequence fragments, such as LEF1 3′UTR-WT and LEF1 3′UTR-Mu, were inserted between the NotI and XhoI cleavage sites of the psiCHECK-2 vector (Promega, Madison, WI, USA) downstream of the *Renilla* luciferase reporter gene. The sequences mentioned above are listed in Additional file 1: Table S1. HEK-293 T cells were seeded into 96-well plates at 70% confluence and then cotransfected with each reporter construct (pmirGLO-LEF1-WT and pmirGLO LEF1-Mu) and Lv-let-7i-3p, Lv-miR-NC, Lv-ASO-let-7i-3p, or LvASO-NC. The Dual-Luciferase Reporter Gene Assay Kit (Beyotime, Shanghai, China) was used to detect the firefly and *Renilla* luciferase activities 48 h after transfection. The firefly values were normalized to *Renilla* luciferase.

### Effect of let-7i-3p on LEF1 in hASCs transfected with the let-7i-3p mimic and inhibitor as determined by immunofluorescence

The fourth-passage hASCs transfected with the let-7i-3p mimic and inhibitor were used to detect the effect of let-7i-3p on LEF1 by immunofluorescence. Two days after transfection, hASCs were seeded on coverslips in 9-well plates. After 48 h, hASCs were fixed with 4% paraformaldehyde for 15 min and washed three times with PBS. Triton X-100 (0.5%, Sigma-Aldrich, USA) was added to each well for 20 min at room temperature. After rinsing three times with PBS, hASCs were blocked with goat serum for 2 h. Primary antibodies specific for LEF1 (1:1000, R&D Systems, USA) were added to the cells and then incubated overnight at 4 °C. Fluorescent Cy3 secondary antibodies (1:50, Proteintech, USA) were added and incubated for 1 h at 37 °C in the dark after rinsing three times with PBS Tween-20. The nucleus was then restained with 4′,6-diamidino-2-phenylindole (DAPI, Sigma-Aldrich, USA). Cells were subsequently viewed by fluorescence microscopy (ZEISS, Oberkochen, Germany).

### Gene expression detection by qPCR

Total RNA was isolated from hASCs by TRIzol Reagent and detected by a NanoVue™ Plus spectrophotometer (GE Healthcare Life Sciences, USA) to determine purity at 260/280 nm. cDNA was synthesized from RNA using PrimeScript RT Master Mix (Perfect Real-Time, TaKaRa, Japan). PCR was performed on the ABI 7300 Real-Time PCR System (Applied Biosystems, UK). The following markers associated with osteogenesis and the Wnt/β-catenin pathway alkaline phosphatase (ALP), runt-related transcription factor 2 (RUNX2), secreted protein acidic and cysteine-rich (SPARC), LEF1, β-catenin, and glyceraldehyde-3-phosphate dehydrogenase (GAPDH). All primer sequences used in this study are shown in Table 2. The results of qPCR are shown as Ct values and compared with GAPDH.

**Table 2:** The primer sequences used for qPCR in this study

Gene	Accession no.	5′-3′	Tm (°C)
RUNX2	NM_001015051	F: TAGATAGTGATTGCGTTTGGCTATG	60
		R: CACTAAGAAATGTTTCAAGGGTCC	60
ALP	NM_003064	F: GAAAGTCCTTCAAAGCTGGAGTCT	60
		R: TCTGGCACTCAGGTTTCTTGTATC	60
SPARC	NM_001309443	F: TGTGATCTAAATCCACTCCTTCCA	60
		R: ACAAACCATCCAAACATTTTAAACA	60
LEF1	NM_001130714	F: TGCCAAATATGAATAACGACCCA	60
		R: GAGAAAAGTGCTCGTCACTGT	60
β-catenin	NM_001098209	F: AAAATGGCAGTGCGTTTAG	60
		R: TTTGAAGGCAGTCTGTCGTA	60
GAPDH	NM_001256799.2	F: GAACGGGAAGCTCACTGG	60
		R: GCCTGCTTCACCACCTTCT	60

### Protein expression detection by western blot

Adherent hASCs were lysed with cell lysis buffer for western blot and immunoprecipitation (IP) analyses (Beyotime, China) and Nuclear and Cytoplasmic Protein Extraction Kit (Beyotime, China) respectively. Then, samples centrifuged at 4 °C (16,000*g*, 20 min). The protein concentrations were determined using a BCA Protein Assay Kit (Beyotime, China). The protein was boiled at 100 °C for 5 min after the addition of 5 × SDS loading buffer (Beyotime, China). Protein samples were loaded onto SDS polyacrylamide gel electrophoresis gels and then electrophoresed at 100 V for 1.5 h. Then, the protein blots were transferred onto membranes (GE Healthcare Life Sciences, USA), which were blocked with TBS containing 5% nonfat milk overnight at 4 °C. Then, the membranes were incubated with primary antibodies specific for ALP (1:1000, Abcam, USA), RUNX2 (1:1000, Abcam, USA), SPARC (1:1000, Abcam, USA), LEF1 (1:1000, R&D Systems, USA), β-catenin (1:1000, Abcam, USA), and GAPDH (1:1000, Cell Signaling Technology, USA) overnight at 4 °C. Afterward, the membranes were washed three times with TBS-0.05% Tween 20 and incubated with the corresponding secondary antibodies for 1 h at room temperature. Next, ECL solution (Thermo Fisher Scientific, Germany) was added to the membranes and incubated for 1 min. The protein blots were visualized by exposure to enhanced chemiluminescence reagents (GE Healthcare, USA). Grayscales on the blots were analyzed by Quantity One software (Bio-Rad, Hercules, CA, USA).

### Statistical analysis

Statistical values were calculated using SPSS 20.0 software (IBM SPSS Statistics, Armonk, NY: IBM-Corp.) One-way analysis of variance (ANOVA) was used for multiple group comparisons. Two-way ANOVA was used for two-group comparisons. All of the results are presented as the mean ± sem, and statistical significance was set at *p* < 0.05.

## Results

### Cell growth and cycle of hASCs

**Fig. 1:**
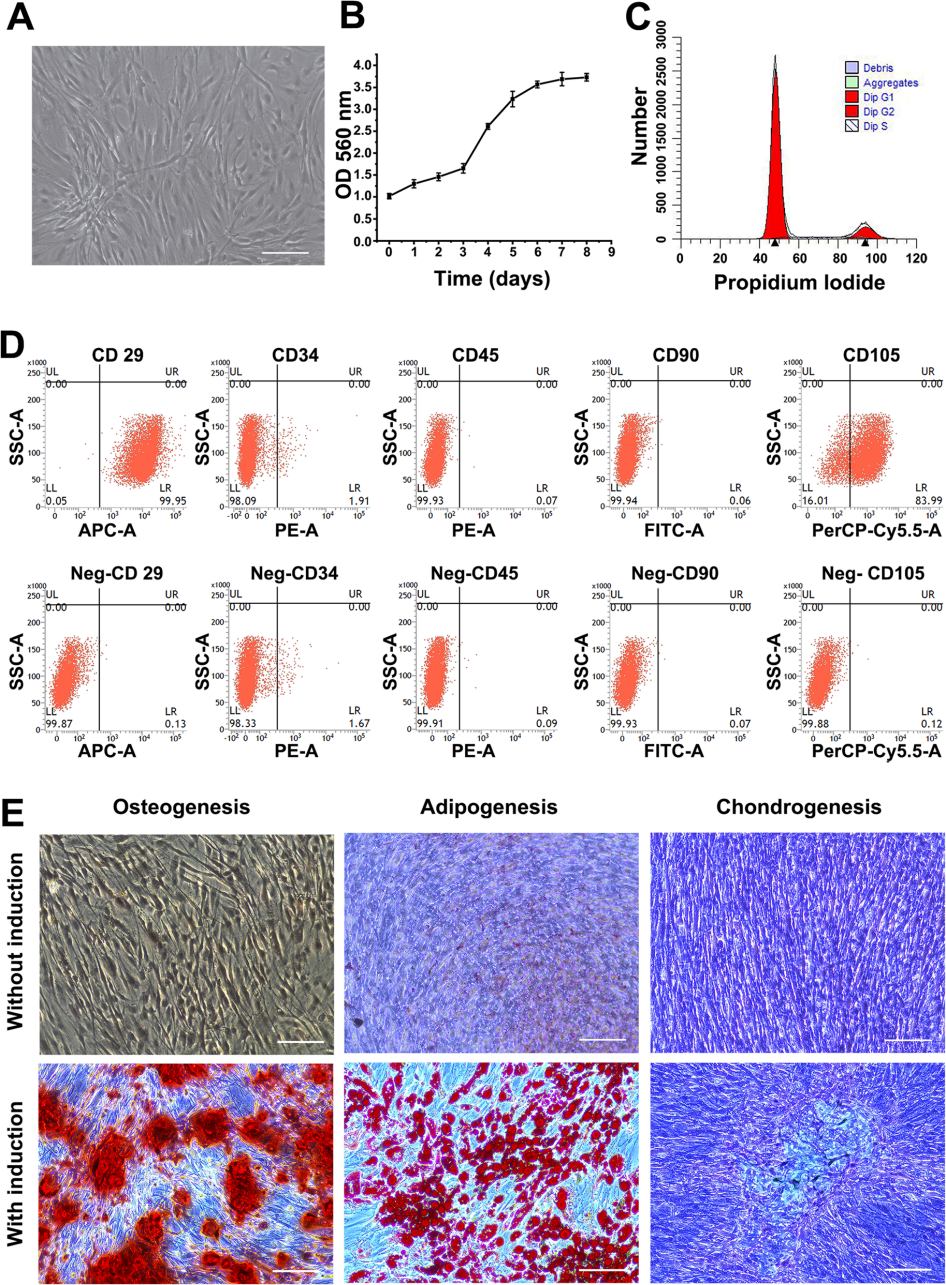
The characteristics of hASCs. **a** The fourth-passage hASCs screened by light microscopy (scale bar 100 μm). **b** The growth curve was determined by CCK-8 assays. hASCs were in the slow growth phase from the first to the third days, exponential growth phase from the third to the sixth days, and the plateau phrase from the seventh d. **c** hASC cell cycle analysis by flow cytometry: G1 phase (82.12% ± 5.83%) and S/G2 phase (17.88% ± 2.59%). **d** Immunophenotypes of hASCs detected by flow cytometry: CD29(+) (99.81% ± 0.76%), CD90(+) (98.43% ± 1.77%), CD105(+) (92.21% ± 6.48%); CD34(−) (2.39% ± 0.72%), CD45(−) (0.97% ± 0.41%). **e** Multilineage of hASCs induced by media. The mineralized nodules were stained with Alizarin Red. The lipid droplets were stained with Oil Red O. The proteoglycans were stained with Alcian Blue (scale bar 100 μm)

As shown by a light microscope (magnification × 100; Leica Microsystems GmbH, Germany), the fourth-passage hASCs had a typical fibroblast-like morphology (Fig. 1a). The growth curve was drawn based on the results of CCK-8 assays (Fig. 1b). In the first 3 days, hASCs were in the slow growth phase; from the third to sixth days, they entered the exponential growth phase; and at the seventh day, they were in the plateau phrase. The cell cycle of hASCs was detected by flow cytometry, revealing that 82.12% ± 5.83% of the cells were in G1 phase, and 17.88% ± 2.59% of the cells were in S/G2 phase (Fig. 1c).

### Immunophenotypes and differentiation of hASCs

Flow cytometry was used to determine the immunophenotypes of hASCs. The cells were positive for CD29 (99.81% ± 0.76%), CD90 (98.43% ± 1.77%), and CD105 (92.21% ± 6.48%) and negative for CD34 (2.39% ± 0.72%) and CD45 (0.97% ± 0.41%) (Fig. 1d).

The mineralized nodules in hASCs, which were cultured in the osteogenic medium after 21 days, were also stained by Alizarin Red (Fig. 1e). The lipid droplets in hASCs, which were cultured in the adipogenic medium after 14 days, were stained by Oil Red O (Fig. 1e). The proteoglycans in hASCs, which were cultured in the adipogenic medium after 14 days, were stained by Alcian Blue to confirm the existence of proteoglycans (Fig. 1e).

### The osteogenic differentiation of hASCs under cyclic strain

The osteogenic differentiation and Wnt/β-catenin pathway of hASCs, which were under cyclic strain for 6 days, were analyzed by qPCR and western blot. All the following results were compared to those obtained using hASCs cultured in α-MEM with 10% FBS at 37 °C and 5% CO_2_ without cyclic strain.

**Fig. 2:**
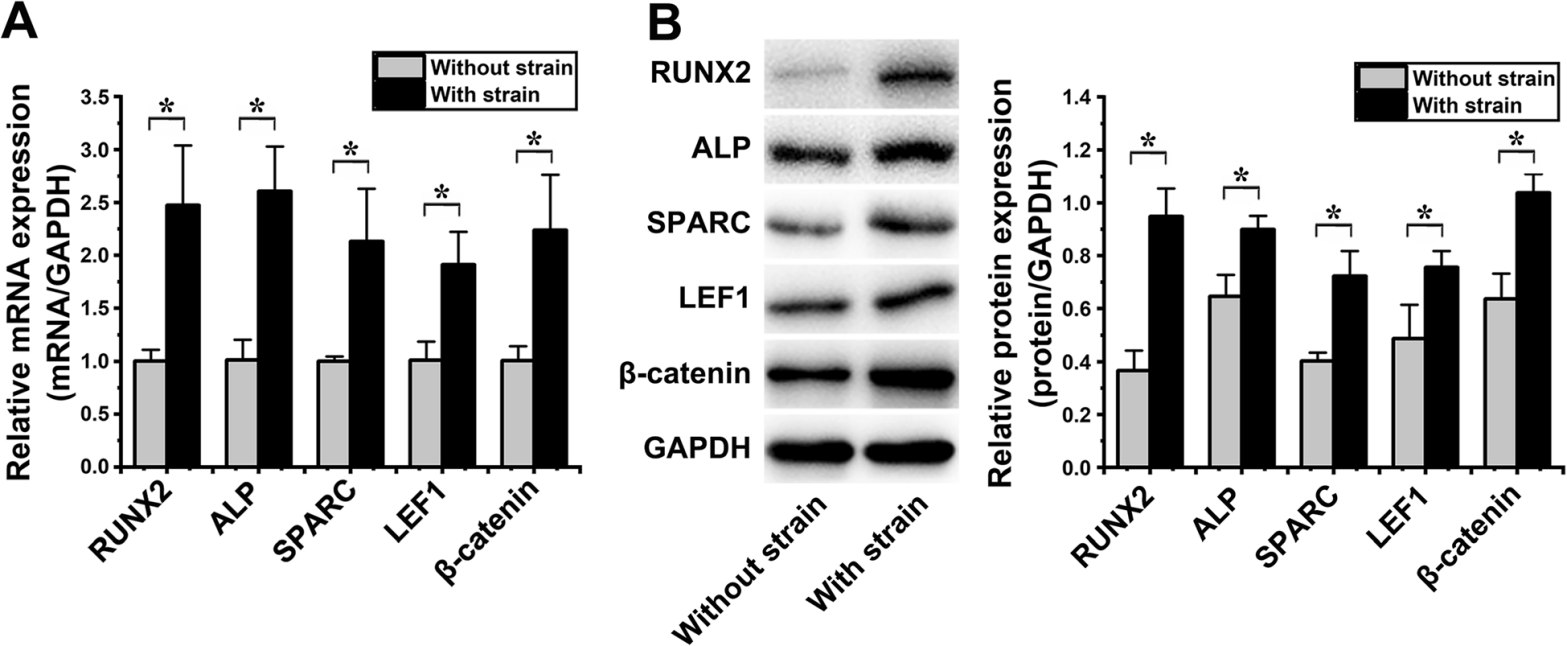
The osteogenic differentiation of hASCs under cyclic strain (*p < 0.05, significant differences existed between these two groups). **a** qPCR results. The mRNA levels of RUNX (22.47 ± 0.73-fold, *p* = 0.011), ALP (2.57 ± 0.09-fold, *p* = 0.004), SPARC (2.13 ± 0.60-fold, *p* = 0.018), LEF1 (1.89 ± 0.45-fold, *p* = 0.012), and β-catenin (2.22 ± 0.51-fold, *p* = 0.017) were significantly increased. **b** Western blot results. The pictures of protein blots are shown in the left column. The protein expression levels of RUNX2 (2.59 ± 0.31-fold, *p* = 0.001), ALP (1.39 ± 0.09-fold, *p* = 0.010), SPARC (1.79 ± 0.21-fold, *p* = 0.005), LEF1 (1.55 ± 0.39-fold, *p* = 0.030), and β-catenin (1.63 ± 0.20-fold, *p* = 0.004) were significantly increased

By qPCR analysis, RUNX2, ALP, and SPARC, which are associated with osteogenic differentiation, were significantly increased 2.47 ± 0.73-fold (*p* = 0.011), 2.57 ± 0.09-fold (*p* = 0.004), and 2.13 ± 0.60-fold (*p* = 0.018), respectively; LEF1 and β-catenin, which are associated with the Wnt/β-catenin pathway, were increased 1.89 ± 0.45-fold (*p* = 0.012) and 2.22 ± 0.51-fold (*p* = 0.017), respectively (Fig. 2a).

Western blot analysis showed that RUNX2, ALP, and SPARC levels were significantly increased 2.59 ± 0.31-fold (*p* = 0.001), 1.39 ± 0.09-fold (*p* = 0.010), and 1.79 ± 0.21-fold (*p* = 0.005), respectively; LEF1 and β-catenin levels were increased 1.55 ± 0.39-fold (*p* = 0.030) and 1.63 ± 0.20-fold (*p* = 0.004), respectively (Fig. 2b).

### Microarray detection of miRNA changes under cyclic strain

**Fig. 3:**
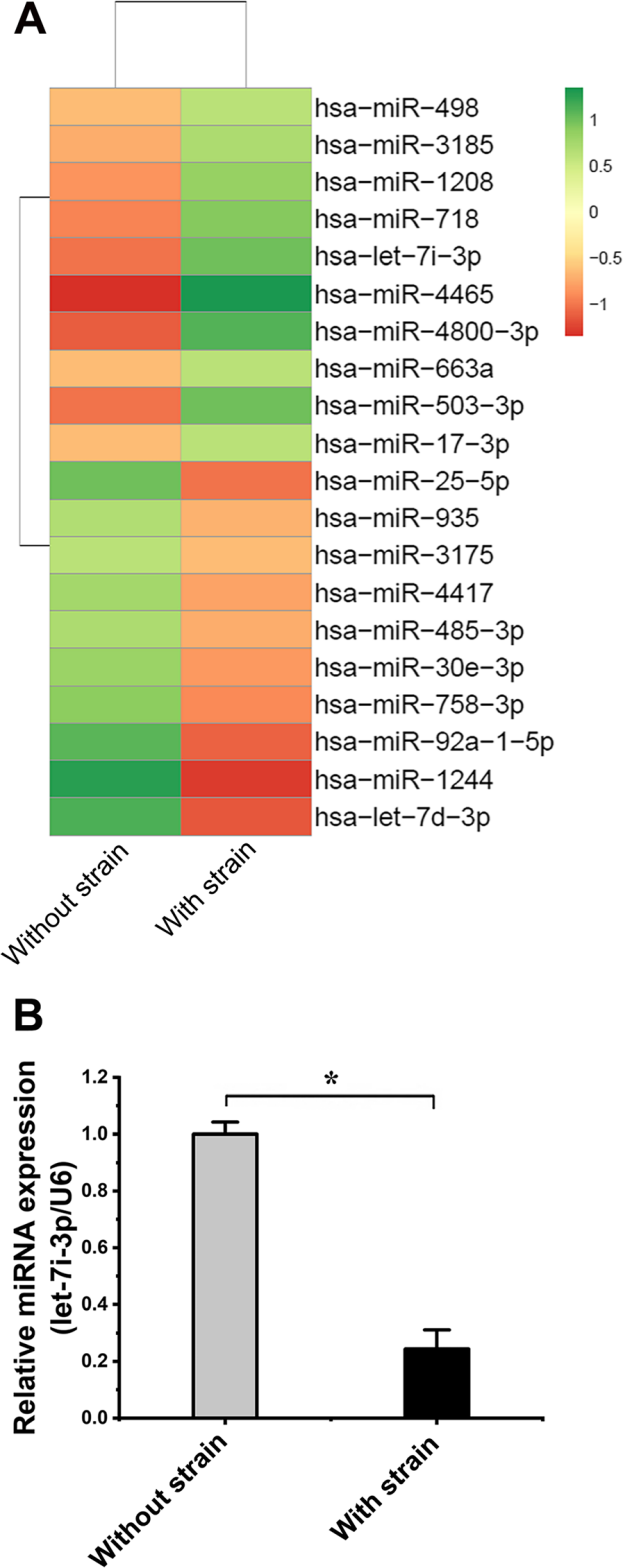
The results of microarray analysis of miRNAs in hASCs under cyclic strain (*p < 0.05, a significant difference existed between these two groups). **a** Twenty miRNAs are listed in the microarray results. Ten miRNAs were upregulated, and 10 were downregulated. They were all significantly changed (> 2-fold). let-7i-3p was reduced 4.11-fold. **b** qPCR was used to confirm the results of let-7i-3p, and the expression of let-7i-3p was reduced 4.10 ± 1.25-fold (*p*<0.000)

After the exertion of cyclic strain on hASCs for 6 days, the microarray analysis of miRNAs showed that the expression levels of 237 miRNAs in hASCs were significantly changed (> 2-fold). Among them, 150 miRNAs exhibited decreased in expression, and 87 miRNAs exhibited increased expression. Some miRNAs associated with Wnt/β-catenin and their fold changes are shown in Fig. 3. The complete microarray results depicting miRNA changes are shown in Additional file 2: Figure S1.

### LEF1 is a target of let-7i-3p

**Fig. 4:**
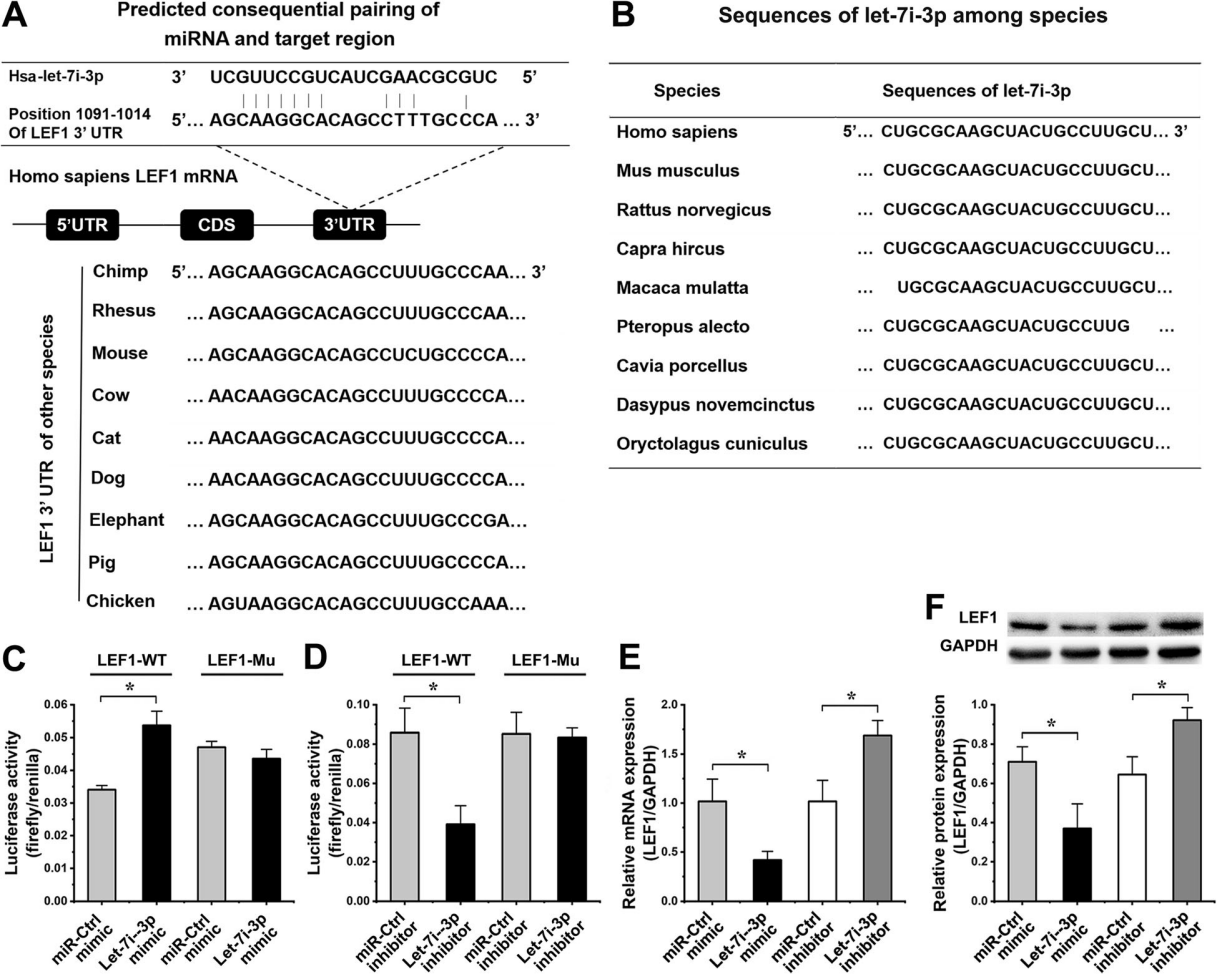
The target of let-7i-3p was LEF1 (*p < 0.05, a significant difference existed between these two groups). **a** The sequence of the LEF1 3′UTR could bind to the sequence of let-7i-3p. The predicted binding sequences of the LEF1 3′UTR were conserved among many species. **b** The sequences of let-7i-3p were conserved among other species. **c** The results of dual-luciferase reporter assays after overexpressing let-7i-3p. The luciferase activity of hASCs cotransfected with the let-7i-3p mimic and LEF1-WT was increased 1.58 ± 0.37-fold (*p* = 0.011) compared with that in the miR-Ctrl inhibitor and LEF1-WT group. The luciferase activity of hASCs cotransfected with the let-7i-3p inhibitor and LEF1-Mu was not significantly different (*p* = 0.15) compared to that in the miR-Ctrl mimic and LEF1-Mu group. **d** The results of dual-luciferase reporter assays after inhibition of let-7i-3p. The luciferase activity of hASCs cotransfected with the let-7i-3p inhibitor and LEF1-WT was decreased 2.19 ± 0.49-fold (*p* = 0.007) compared with that in the miR-Ctrl mimic and LEF1-WT group. The luciferase activity of hASCs cotransfected with the let-7i-3p mimic and LEF1-WT was not significantly different (*p* = 0.81) from that in the miR-Ctrl mimic and LEF1-WT group. **e** LEF1 expression in hASCs after overexpressing or inhibiting let-7i-3p was analyzed by qPCR. LEF1 mRNA levels were decreased 2.43 ± 0.24-fold (*p* = 0.013) in hASCs transfected with the let-7i-3p mimic compared to that in the miR-Ctrl mimic group and increased 1.66 ± 0.07-fold (*p* = 0.002) in hASCs transfected with the let-7i-3p inhibitor compared to that in the miR-Ctrl inhibitor group. **f** LEF1 expression in hASCs after overexpressing or inhibiting let-7i-3p was analyzed by western blot. The LEF1 protein level was decreased 1.92 ± 0.63-fold (*p* = 0.016) in hASCs transfected with let-7i-3p mimic compared to that in the miR-Ctrl mimic group and increased 1.43 ± 0.10-fold (*p* = 0.013) in hASCs transfected with the let-7i-3p inhibitor compared to that in the miR-Ctrl inhibitor group

The predicted binding sequences of both the 3′UTR in LEF1 and let-7i-3p are highly conserved across species (Fig. 4a, b). The LEF1 3′UTR, which is a component of the Wnt/β-catenin signaling pathway, was likely matched to let-7i-3p (Fig. 4a).

Overexpression of let-7i-3p significantly inhibited the luciferase activity of pMIR/LEF1-WT reporter genes, and the luciferase activity was decreased by 2.19 ± 0.49-fold (*p* = 0.007) compared with that in the control group, which was a significant difference. In the reporter gene system in which the LEF1 mRNA 3′UTR binding sites were mutated, overexpression of let-7i-3p did not affect the reporter gene luciferase activity. Inhibition of let-7i-3p expression induced the luciferase activity of the reporter gene. Compared with that in the control group, the luciferase activity in the let-7i-3p inhibition group was increased 1.58 ± 0.37-fold (*p* = 0.011), and the difference was significant. Then, combination with the LEF1 mRNA 3′UTR was assessed. In the reporter gene system with a site mutation, inhibition of let-7i-3p expression did not affect the reporter gene luciferase activity. The results are shown in Fig. 4 c and d.

LEF1 mRNA expression in hASCs transfected with let-7i-3p mimic was decreased 2.43 ± 0.24-fold (*p* = 0.013), as shown by qPCR, compared to that in hASCs transfected with the miR-Ctrl mimic. LEF1 mRNA expression in hASCs transfected with the let-7i-3p inhibitor was increased 1.66 ± 0.07-fold (*p* = 0.002), as shown by qPCR, compared to that in hASCs transfected with the miR-Ctrl inhibitor (Fig. 4e). LEF1 protein expression in hASCs transfected with let-7i-3p mimic was decreased 1.92 ± 0.63-fold (*p* = 0.016), as shown by western blot, compared to that in hASCs transfected with the miR-Ctrl mimic. LEF1 protein expression in hASCs transfected with the let-7i-3p inhibitor was increased 1.43 ± 0.10-fold (*p* = 0.013), as shown by western blot, compared to that in hASCs transfected with the miR-Ctrl inhibitor (Fig. 4f). Together, these results suggest that LEF1 is a target gene of let-7i-3p.

### Immunofluorescence analysis of hASCs transfected with let-7i-3p mimics and inhibitor

**Fig. 5:**
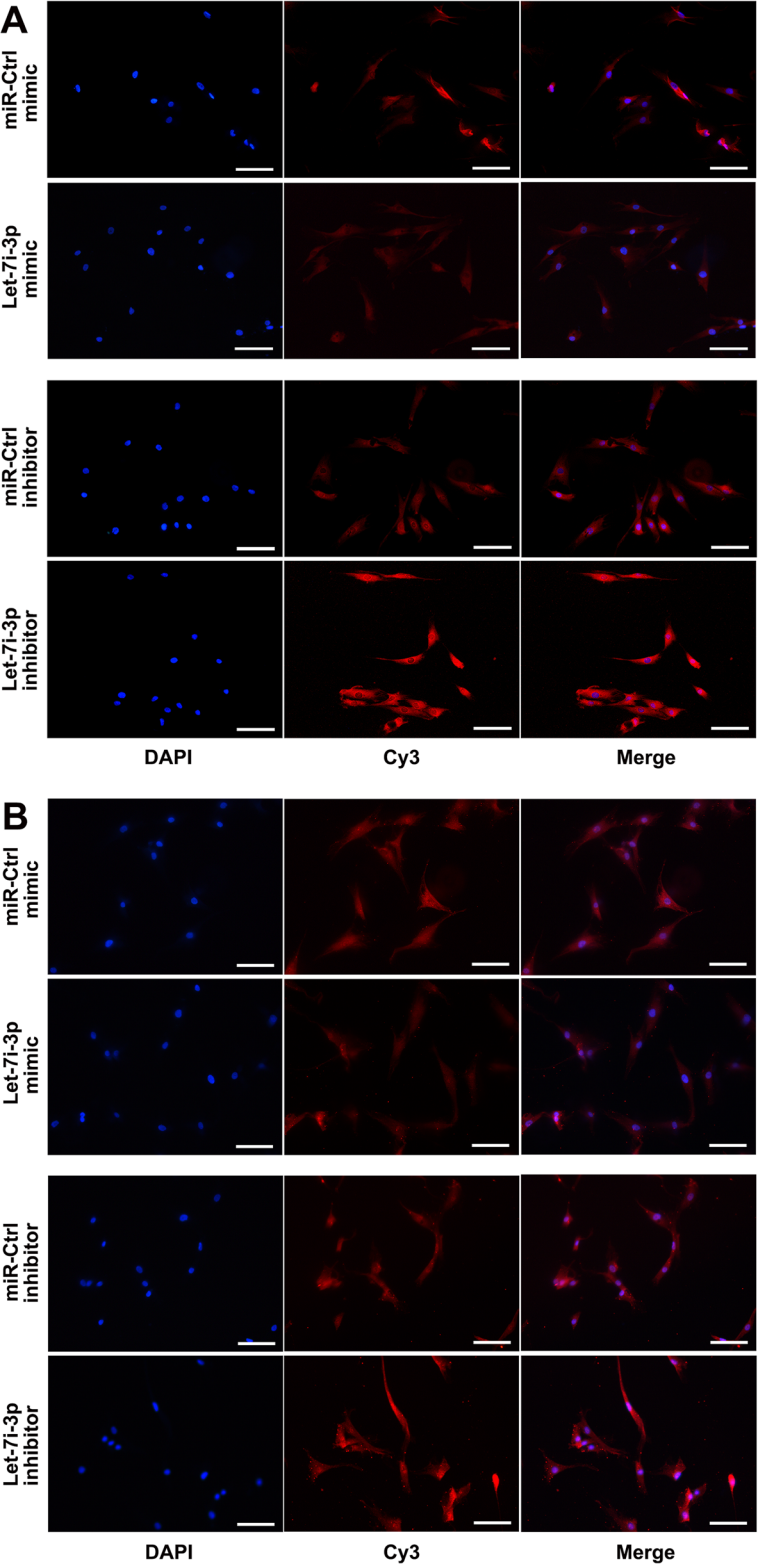
Immunofluorescence analysis of hASCs transfected with let-7i-3p mimics and inhibitor. **a** Compared to that in normal cultured hASCs, the immunofluorescence intensity of LEF1 was decreased in hASCs transfected with let-7i-3p mimics. Compared with that in normal cultured hASCs, the immunofluorescence intensity of LEF1 was increased in hASCs transfected with the let-7i-3p inhibitor. **b** Compared to that in normal cultured hASCs, the immunofluorescence intensity of β-catenin was decreased in both the cytoplasm and nucleus of hASCs transfected with let-7i-3p mimics. Compared to that in normal cultured hASCs, the immunofluorescence intensity of β-catenin in both the cytoplasm and nucleus was promoted after transfection of the let-7i-3p inhibitor (scale bar 100 μm)

The immunofluorescence intensity of LEF1 and β-catenin in hASCs was inhibited after transfection of let-7i-3p mimics compared with that in normal cultured hASCs (Fig. 5). The immunofluorescence intensity of LEF1 and β-catenin in hASCs was promoted after transfection of the let-7i-3p inhibitor compared with that in normal cultured hASCs (Fig. 5a). The immunofluorescence intensity of β-catenin in both the cytoplasm and nucleus of hASCs was promoted after transfection of the let-7i-3p inhibitor compared with that in normal cultured hASCs (Fig. 5b).

### Effect of LEF1 on the osteogenic differentiation of hASCs under cyclic strain

**Fig. 6:**
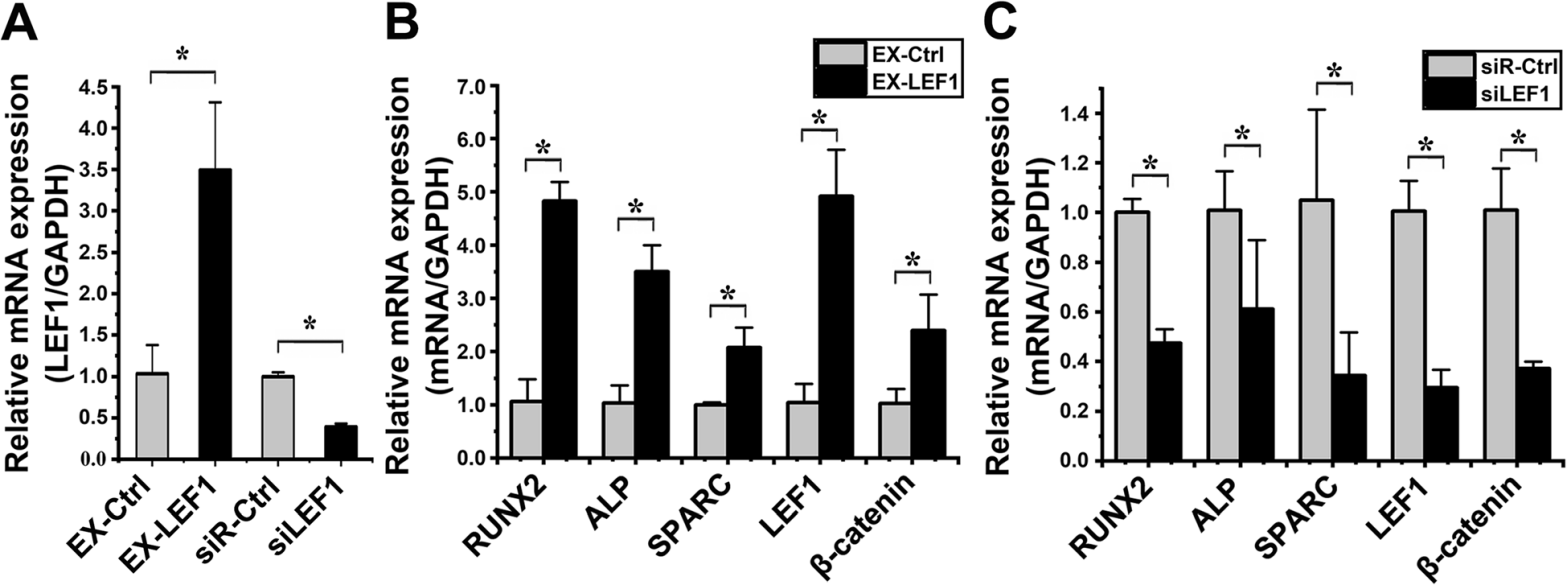
Modulation of LEF1 on the osteogenic differentiation of hASCs under cyclic strain (*p < 0.05, significant differences existed between these two groups). **a** Transfection efficiency detected by qPCR. The expression of LEF1 in the EX-LEF1 group was increased 3.37 ± 1.24-fold (*p* = 0.009) compared to that in the EX-Ctrl group. The expression of LEF1 in the siLEF1 group was significantly decreased 2.56 ± 0.03-fold (*p* < 0.001) compared to that in the siR-Ctrl group. **b** The effect of LEF1 overexpression on the osteogenic differentiation of hASCs under cyclic strain as determined by qPCR. Compared to those in the EX-Ctrl group, the levels of the following mRNA markers in hASCs transfected with EX-LEF1 under cyclic strain for 6 days were significantly increased: RUNX2 (4.54 ± 2.64-fold, *p* < 0.001), ALP (3.38 ± 0.64-fold, *p* = 0.002), SPARC (2.07 ± 0.35-fold, *p* = 0.008), LEF1 (4.67 ± 0.47-fold, *p* = 0.008), and β-catenin (2.33 ± 0.39-fold, *p* = 0.023). **c** Effect of LEF1 suppression on the osteogenic differentiation of hASCs under cyclic strain as determined by qPCR. Compared to those in the miR-Ctrl inhibitor group, the levels of the following mRNA markers in hASCs transfected with EX-LEF1 under cyclic strain for 6 days were significantly decreased: RUNX (2.11 ± 0.33-fold, *p* < 0.001), ALP (1.65 ± 0.68-fold, *p* = 0.044), SPARC (3.04 ± 1.38-fold, *p* = 0.039), LEF1 (3.40 ± 1.18-fold, *p* = 0.001), and β-catenin (2.72 ± 0.44-fold, *p* = 0.003)

To determine the transfection efficiency, the expression of LEF1 was detected by qPCR after transfection of EX-LEF1, siLEF1, EX-Ctrl, and siR-Ctrl into hASCs. Compared to that in the EX-Ctrl group, the LEF1 expression in the EX-LEF1 group was increased 3.37 ± 1.24-fold (*p* = 0.009) (Fig. 6a). Compared to that in the siR-Ctrl group, the LEF1 expression in the siLEF1 group was significantly decreased 2.56 ± 0.19-fold (*p* < 0.001) (Fig. 6a).

qPCR analysis revealed the effect of LEF1 on the osteogenic differentiation of hASCs under cyclic strain. Compared to those in the EX-Ctrl group, the RUNX2, ALP, SPARC, LEF1, and β-catenin levels in hASCs transfected with EX-LEF1 and then exposed to loading cyclic strain for 6 days were significantly increased 4.54 ± 2.64-fold (*p* < 0.001), 3.38 ± 0.64-fold (*p* = 0.002), 2.07 ± 0.35-fold (*p* = 0.008), 4.67 ± 0.47-fold (*p* = 0.008), and 2.33 ± 0.39-fold (*p* = 0.023), respectively (Fig. 6b). Compared to those in the siR-Ctrl group, the RUNX2, ALP, SPARC, LEF1 and β-catenin levels in hASCs transfected with siLEF1 and then exposed to loading cyclic strain for 6 days were decreased 2.11 ± 0.33-fold (*p* < 0.001), 1.65 ± 0.68-fold (*p* = 0.044), 3.04 ± 1.38-fold (*p* = 0.039), 3.40 ± 1.18-fold (*p* = 0.001), and 2.72 ± 0.44-fold (*p* = 0.003), respectively (Fig. 6c).

As shown by the above results, the osteogenic differentiation of hASCs was induced by overexpression of LEF1. In contrast, the osteogenic differentiation of hASCs was inhibited by knockdown of LEF1.

### Effect of β-catenin on the osteogenic differentiation of hASCs under cyclic strain

**Fig. 7:**
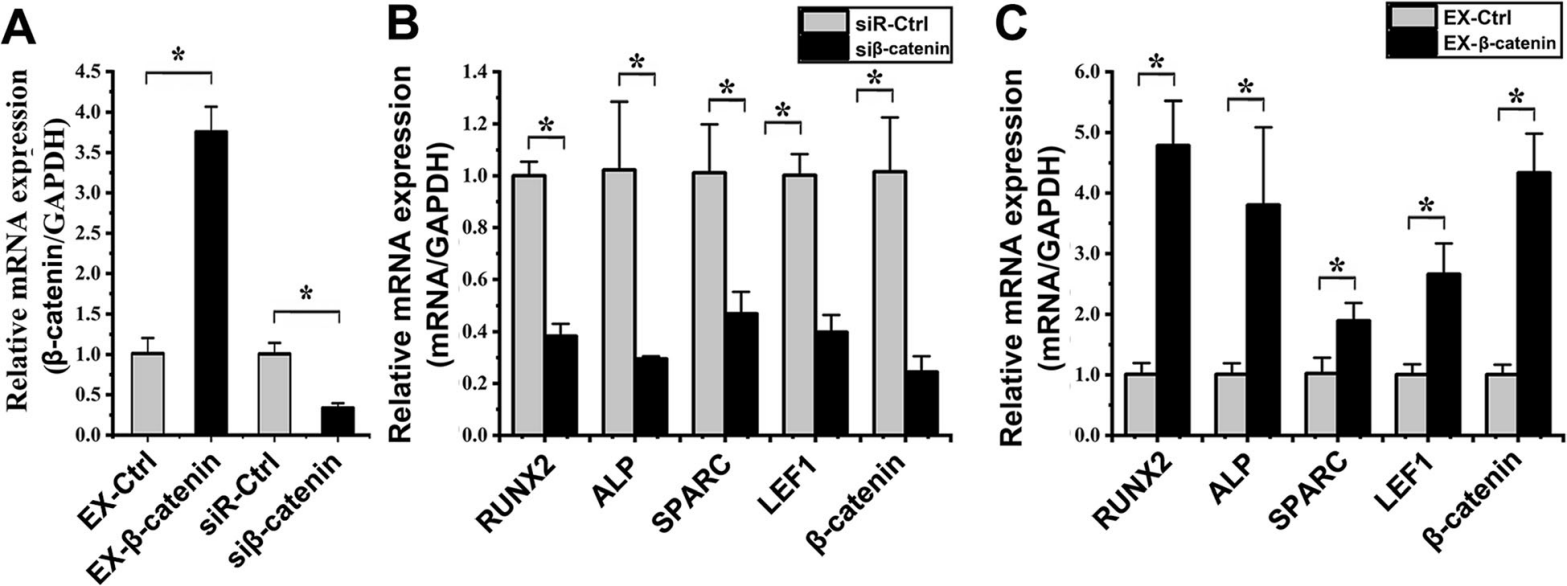
Effects of β-catenin modulation on the osteogenic differentiation of hASCs under cyclic strain (*p < 0.05, significant differences existed between these two groups). **a** Transfection efficiency detected by qPCR. The expression of β-catenin in the EX-β-catenin group was increased 3.71 ± 0.42-fold (*p* < 0.001) compared to that in the EX-Ctrl group. The expression of β-catenin in the siβ-catenin group was significantly decreased 3.00 ± 0.76-fold (*p* = 0.002) compared to that in the siR-Ctrl group. **b** The effect of β-catenin overexpression on the osteogenic differentiation of hASCs under cyclic strain as determined by qPCR. Compared to those in the EX-Ctrl group, the levels of the following mRNA markers in hASCs transfected with EX-β-catenin under cyclic strain for 6 days were significantly increased: RUNX2 (4.73 ± 1.60-fold, *p* = 0.001), ALP (3.76 ± 2.04-fold, *p* = 0.021), SPARC (1.85 ± 0.49-fold, *p* = 0.004), LEF1 (2.64 ± 0.40-fold, *p* = 0.006), and β-catenin (4.30 ± 1.12-fold, *p* = 0.001). **c** The effect of β-catenin suppression on the osteogenic differentiation of hASCs under cyclic strain as determined by qPCR. Compared to those in the miR-Ctrl inhibitor group, the levels of the following mRNA markers in hASCs transfected with EX-β-catenin under cyclic strain for 6 days were significantly decreased: RUNX (2.61 ± 0.45-fold, *p* < 0.001), ALP (3.46 ± 0.79-fold, *p* = 0.009), SPARC (2.16 ± 0.02-fold, *p* = 0.010), LEF1 (2.52 ± 0.61-fold, *p* = 0.001), and β-catenin (4.15 ± 1.72-fold, *p* = 0.004)

To determine the transfection efficiency, the expression of β-catenin was detected by qPCR after transfection of EX-β-catenin, siβ-catenin, EX-Ctrl, and siR-Ctrl into hASCs. Compared to that in the EX-Ctrl group, the β-catenin expression in the EX-β-catenin group was increased 3.71 ± 0.42-fold (*p* < 0.001) (Fig. 7a). Compared to that in the siR-Ctrl group, the β-catenin expression in the siβ-catenin group was significantly decreased 3.00 ± 0.76-fold (*p* = 0.002) (Fig. 7a).

qPCR analysis revealed the effect of β-catenin on the osteogenic differentiation of hASCs under cyclic strain. Compared to those in the EX-Ctrl group, the levels of RUNX2, ALP, SPARC, LEF1, and β-catenin in hASCs transfected with EX-β-catenin and then exposed to loading cyclic strain for 6 days were significantly increased 4.73 ± 1.60-fold (*p* = 0.001), 3.76 ± 2.04-fold (*p* = 0.021), 1.85 ± 0.49-fold (*p* = 0.004), 2.64 ± 0.40-fold (*p* = 0.006), and 4.30 ± 1.12-fold (*p* = 0.001), respectively (Fig. 7b). Compared to those in the siR-Ctrl group, the levels of RUNX2, ALP, SPARC, LEF1, and β-catenin in hASCs transfected with siβ-catenin and then exposed to loading cyclic strain for 6 days were decreased 2.61 ± 0.45-fold (*p* < 0.001), 3.46 ± 0.79-fold (*p* = 0.009), 2.16 ± 0.02-fold (*p* = 0.010), 2.52 ± 0.61-fold (*p* = 0.001), and 4.15 ± 1.72-fold (*p* = 0.004), respectively (Fig. 7c).

As shown by the above results, the osteogenic differentiation of hASCs was induced by overexpression of β-catenin. In contrast, the osteogenic differentiation of hASCs was inhibited by knockdown of β-catenin.

### Effects of let-7i-3p on the osteogenic differentiation of hASCs under cyclic strain

**Fig. 8:**
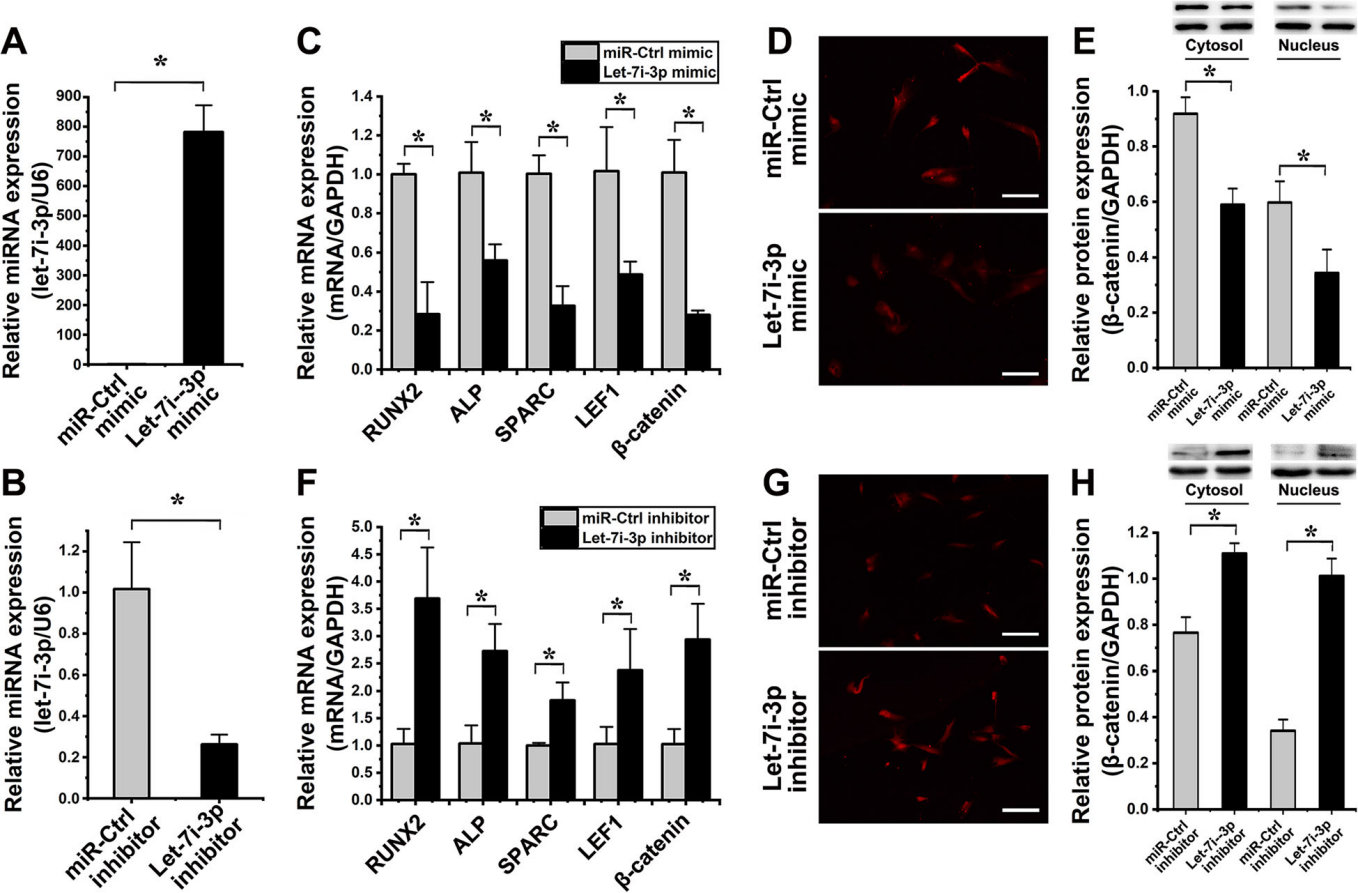
Let-7i-3p modulates the osteogenic differentiation of hASCs under cyclic strain (*p < 0.05, significant differences existed between these two groups). **a** Transfection efficiency detected by qPCR. The expression of let-7i-3p in hASCs transfected with the let-7i-3p mimic was increased 776.40 ± 111.78-fold (*p* < 0.001). **b** The expression of let-7i-3p in hASCs transfected with the let-7i-3p inhibitor was decreased 3.88 ± 0.11-fold (*p* = 0.005). **c** The effect of let-7i-3p overexpression on the osteogenic differentiation of hASCs under cyclic strain as determined by qPCR. Compared to those in the miR-Ctrl mimic group, the levels of the following mRNA markers in hASCs transfected with the let-7i-3p mimic were significantly decreased: RUNX2 (3.52 ± 1.94-fold, *p* = 0.002), ALP (1.80 ± 0.15-fold, *p* = 0.012), SPARC (3.07 ± 0.86-fold, *p* < 0.001), LEF1 (2.08 ± 0.73-fold, *p* = 0.018), and β-catenin (3.60 ± 0.57-fold, *p* = 0.002). **d** The immunofluorescence intensity of β-catenin in the nucleus was weakened in hASCs transfected with the let-7i-3p mimic (scale bar 100 μm). **e** The expression of cytoplasmic and nuclear β-catenin in hASCs transfected with the let-7i-3p mimic significantly respectively decreased 1.56 ± 0.06-fold (*p* = 0.002) and 1.74 ± 0.24-fold (*p* = 0.019) than those in the miR-Ctrl mimic group. **f** The effect of let-7i-3p suppression on the osteogenic differentiation of hASCs under cyclic strain as determined by qPCR. Compared to those in the miR-Ctrl inhibitor group, the levels of the following mRNA markers in hASCs transfected with the let-7i-3p inhibitor were significantly increased: RUNX2 (3.58 ± 0.82-fold, *p* = 0.009), ALP (2.53 ± 0.49-fold, *p* = 0.008), SPARC (1.83 ± 0.28-fold, *p* = 0.012), LEF1 (2.31 ± 1.03-fold, *p* = 0.046), and β-catenin (2.86 ± 1.68-fold, *p* = 0.010). **g** The immunofluorescence intensity of β-catenin in the nucleus was enhanced in hASCs transfected with the let-7i-3p inhibitor (scale bar 100 μm). **h** The expression of cytoplasmic and nuclear β-catenin in hASCs transfected with the let-7i-3p inhibitor significantly respectively increased 1.45 ± 0.11-fold (*p* = 0.003) and 2.97 ± 0.19-fold (*p* = 0.001) than those in the miR-Ctrl inhibitor group

To determine the transfection efficiency, the expression of let-7i-3p in hASCs was detected by qPCR after transfection with the let-7i-3p mimic and inhibitor, miR-Ctrl mimic, and miR-Ctrl inhibitor. Compared to that in the miR-Ctrl mimic group, the expression of let-7i-3p in the let-7i-3p mimic group was increased 776.40 ± 111.78-fold (*p* < 0.001) (Fig. 8a). Compared to that in the miR-Ctrl inhibitor group, the expression of let-7i-3p in the let-7i-3p inhibitor group was decreased 3.88 ± 1.36-fold (*p* = 0.005) (Fig. 8b).

qPCR analysis revealed the effect of let-7i-3p on the osteogenic differentiation of hASCs under cyclic strain. Compared to those in the miR-Ctrl mimic group, the levels of RUNX2, ALP, SPARC, LEF1, and β-catenin in hASCs transfected with the let-7i-3p mimic and then exposed to loading cyclic strain for 6 days were significantly decreased 3.52 ± 1.94-fold (*p* = 0.002), 1.80 ± 0.15-fold (*p* = 0.012), 3.07 ± 0.86-fold (*p* < 0.001), 2.08 ± 0.73-fold (*p* = 0.018), and 3.60 ± 0.57-fold (*p* = 0.002), respectively (Fig. 8c). The immunofluorescence intensity of β-catenin in the hASC nucleus was also decreased in hASCs transfected with the let-7i-3p mimic (Fig. 8d). Compared to those in the miR-Ctrl mimic group, the levels of cytoplasmic and nuclear β-catenin in hASCs transfected with the let-7i-3p mimic significantly decreased 1.56 ± 0.06-fold (*p* = 0.002) and 1.74 ± 0.24-fold (*p* = 0.019), respectively (Fig. 8e).

Compared to those in the miR-Ctrl inhibitor group, the levels of RUNX2, ALP, SPARC, LEF1, and β-catenin in hASCs transfected with the let-7i-3p inhibitor and then exposed to loading cyclic strain for 6 days were increased 3.58 ± 0.82-fold (*p* = 0.009), 2.53 ± 0.49-fold (*p* = 0.008), 1.83 ± 0.28-fold (*p* = 0.012), 2.31 ± 1.03-fold (*p* = 0.046), and 2.86 ± 1.68-fold (*p* = 0.010), respectively (Fig. 8f). The immunofluorescence intensity of β-catenin in the hASC nucleus was also increased in hASCs transfected with the let-7i-3p inhibitor (Fig. 8g). Compared to those in the miR-Ctrl inhibitor group, the levels of cytoplasmic and nuclear β-catenin in hASCs transfected with the let-7i-3p inhibitor significantly increased 1.45 ± 0.11-fold (*p* = 0.003) and 2.97 ± 0.19-fold (*p* = 0.001), respectively (Fig. 8h).

As shown by the above results, the osteogenic differentiation of hASCs was promoted by the suppression of let-7i-3p. In contrast, the osteogenic differentiation of hASCs was inhibited by overexpression of let-7i-3p.

### Effect of LEF1 regulated by let-7i-3p on the osteogenic differentiation of hASCs under cyclic strain

**Fig. 9:**
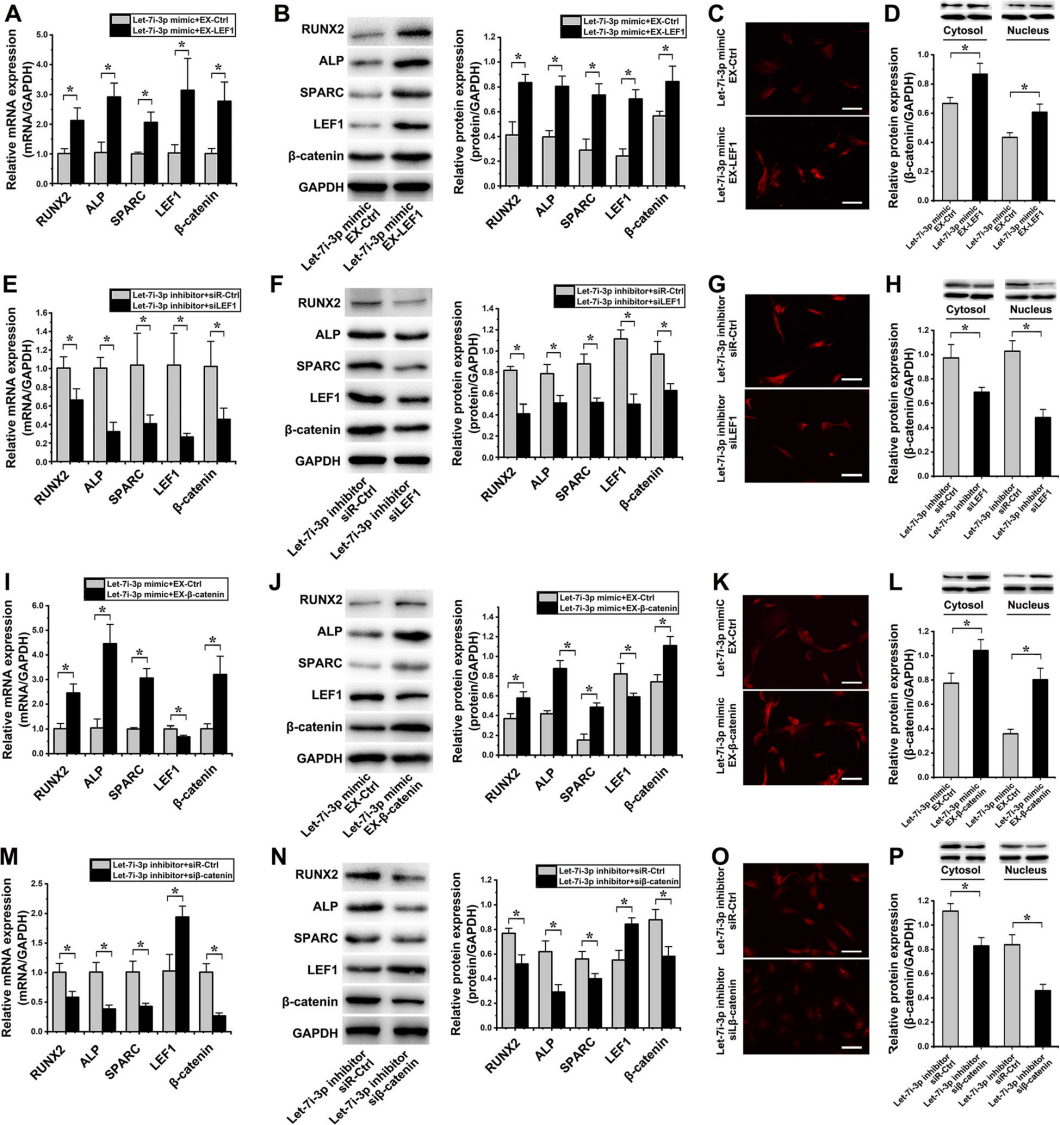
Effect of LEF1 regulated by let-7i-3p on the osteogenic differentiation of hASCs under cyclic strain (*p < 0.05, significant differences existed between these two groups). **a** hASCs were cotransfected with the let-7i-3p mimic and EX-LEF1 under cyclic strain for 6 days, and the levels of the following mRNAs were significantly increased compared to those in hASCs cotransfected with let-7i-3p mimic and EX-Ctrl: RUNX2, ALP, SPARC, LEF1, and β-catenin (2.10 ± 0.69-fold, *p* = 0.014; 2.80 ± 1.62-fold, *p* = 0.005; 2.06 ± 0.30-fold, *p* = 0.006; 3.06 ± 0.52-fold, *p* = 0.029; and 2.75 ± 0.68-fold, *p* = 0.010, repectively). **b** Protein blots are listed in the left column. hASCs were cotransfected with the let-7i-3p mimic and EX-LEF1 under cyclic strain for 6 days. The protein expression levels of the above markers were also increased: RUNX (22.02 ± 0.38-fold, *p* = 0.004), ALP (2.03 ± 0.07-fold, *p* = 0.002), SPARC (2.54 ± 0.60-fold, *p* = 0.004), LEF1 (2.91 ± 0.61-fold, *p* = 0.004), and β-catenin (1.49 ± 0.17-fold, *p* = 0.022). **c** The immunofluorescence intensity of β-catenin in the nucleus was enhanced in hASCs cotransfected with the let-7i-3p mimic and EX-LEF1(scale bar 100 μm). **d** The protein expression levels of cytoplasmic and nuclear β-catenin in hASCs cotransfected with the let-7i-3p mimic and EX-LEF1 were respectively increased 1.30 ± 0.10-fold (*p* = 0.026) and 1.40 ± 0.15-fold (*p* = 0.017) than those in the group transfected with the let-7i-3p mimic and EX-Ctrl. **e** hASCs were cotransfected with the let-7i-3p inhibitor and siLEF1 under cyclic strain for 6 days. The mRNA levels of the above markers were decreased by 1.52 ± 0.26-fold (*p* = 0.026), 3.12 ± 1.55-fold (*p* = 0.002), 2.55 ± 0.25-fold (*p* = 0.038), 3.92 ± 0.72-fold (*p* = 0.018), and 2.25 ± 1.24-fold (*p* = 0.030), respectively, compared to those in hASCs cotransfected with the let-7i-3p inhibitor and siR-Ctrl. **f** Protein blots are listed in the left column. hASCs were cotransfected with the let-7i-3p inhibitor and siLEF1 under cyclic strain for 6 days. The protein expression levels of the above markers were also decreased 2.00 ± 0.42-fold (*p* = 0.002), 1.54 ± 0.17-fold (*p* = 0.013), 1.71 ± 0.19-fold (*p* = 0.003), 2.24 ± 0.28-fold (*p* = 0.001), and 1.55 ± 0.06-fold (*p* = 0.012), respectively. **g** The immunofluorescence intensity of β-catenin in the hASC nucleus was weakened in hASCs cotransfected with the let-7i-3p inhibitor and siβ-catenin (scale bar 100 μm). **h** The protein expression levels of cytoplasmic and nuclear β-catenin in hASCs cotransfected with the let-7i-3p inhibitor and siLEF1were respectively decreased 1.41 ± 0.19-fold (*p* = 0.036) and 2.13 ± 0.35-fold (*p* = 0.001) than those in the group transfected with the let-7i-3p inhibitor and siR-Ctrl. **i** hASCs were cotransfected with the let-7i-3p mimic and EX-β-catenin under cyclic strain for 6 days: compared to those in the group transfected with the let-7i-3p mimic and EX-Ctrl, the mRNA expression levels of RUNX2, ALP, SPARC, and β-catenin were increased 2.42 ± 0.92-fold (*p* = 0.004), 4.27 ± 1.78-fold (*p* = 0.002), 3.06 ± 0.41-fold (*p* = 0.001), and 3.17 ± 1.09-fold (*p* = 0.008); the mRNA expression levels of LEF1 was decreased 1.47 ± 0.21-fold (*p* = 0.016), respectively. **j** Protein blots are listed in the left column. hASCs were cotransfected with the let-7i-3p mimic and EX-β-catenin under cyclic strain for 6 days: compared to those in the group transfected with the let-7i-3p mimic and EX-Ctrl, the protein expression levels of RUNX2, ALP, SPARC and β-catenin were increased 1.57 ± 0.06-fold (*p* = 0.011), 2.10 ± 0.03-fold (*p* = 0.001), 3.16 ± 1.01-fold (*p* = 0.002), and 1.50 ± 0.04-fold (*p* = 0.006); the protein expression levels of LEF1 were decreased 1.40 ± 0.13-fold (*p* = 0.023), respectively. **k** The immunofluorescence intensity of β-catenin in the hASC nucleus was enhanced in hASCs cotransfected with the let-7i-3p mimics and EX-β-catenin (scale bar 100 μm). **l** The protein expression levels of cytoplasmic and nuclear β-catenin in hASCs cotransfected with the let-7i-3p mimic and EX-β-catenin were respectively increased 1.35 ± 0.12-fold (*p* = 0.019) and 2.23 ± 0.27-fold (*p* = 0.008) than those in the group transfected with the let-7i-3p mimic and EX-Ctrl. **m** hASCs were cotransfected with the let-7i-3p mimic and EX-β-catenin under cyclic strain for 6 days: compared to those in the group transfected with the let-7i-3p inhibitor and siβ-catenin, compared to those in the group transfected with the let-7i-3p inhibitor and siR-Ctrl, the mRNA levels of RUNX2, ALP, SPARC, and β-catenin were decreased 1.73 ± 0.24-fold (*p* = 0.015), 2.63 ± 0.69-fold (*p* = 0.004), 2.36 ± 0.46-fold (*p* = 0.006), and 3.75 ± 0.21-fold (*p* = 0.001); the mRNA expression levels of LEF1 was increased 1.89 ± 0.74-fold (*p* = 0.009), respectively. **n** Protein blots are listed in the left column. hASCs were cotransfected with the let-7i-3p mimic and EX-β-catenin under cyclic strain for 6 days: compared to those in the group transfected with the let-7i-3p inhibitor and siR-Ctrl, the protein expression levels of RUNX2, ALP, SPARC, and β-catenin were decreased 1.48 ± 0.15-fold (*p* = 0.007), 2.12 ± 0.14-fold (*p* = 0.006), 1.40 ± 0.01-fold (*p* = 0.020), and 1.51 ± 0.17-fold (*p* = 0.011); the protein expression levels of LEF1 were increased 1.53 ± 0.19-fold (*p* = 0.006), respectively. **o** The immunofluorescence intensity of β-catenin in the hASC nucleus was weaken in hASCs cotransfected with the let-7i-3p inhibitor and siβ-catenin (scale bar 100 μm). **p** The protein expression levels of cytoplasmic and nuclear β-catenin in hASCs cotransfected with the let-7i-3p inhibitor and siβ-catenin were respectively decreased 1.35 ± 0.09-fold (*p* = 0.006) and 1.83 ± 0.04-fold (*p* = 0.004) than those in the group transfected with the let-7i-3p inhibitor and siR-Ctrl

Compared to those in the group transfected with the let-7i-3p mimic and EX-Ctrl, the mRNA expression levels of RUNX2, ALP, SPARC, LEF1, and β-catenin were increased 2.10 ± 0.69-fold (*p* = 0.014), 2.80 ± 1.62-fold (*p* = 0.005), 2.06 ± 0.30-fold (*p* = 0.006), 3.06 ± 0.52-fold (*p* = 0.029), and 2.75 ± 0.68-fold (*p* = 0.010), respectively, in hASCs cotransfected with the let-7i-3p mimic and EX-LEF1 (Fig. 9a); the protein expression levels of the above markers were also increased 2.02 ± 0.38-fold (*p* = 0.004), 2.03 ± 0.07-fold (*p* = 0.002), 2.54 ± 0.60-fold (*p* = 0.004), 2.91 ± 0.61-fold (*p* = 0.004), and 1.49 ± 0.17-fold (*p* = 0.022), respectively (Fig. 9b). The immunofluorescence intensity of β-catenin in the hASC nucleus was also increased in hASCs cotransfected with the let-7i-3p mimic and EX-LEF1 (Fig. 9c). Compared to those in the group transfected with the let-7i-3p mimic and EX-Ctrl, the protein expression levels of cytoplasmic and nuclear β-catenin were increased 1.30 ± 0.10-fold (*p* = 0.026) and 1.40 ± 0.15-fold (*p* = 0.017), respectively, in hASCs cotransfected with the let-7i-3p mimic and EX-LEF1 (Fig. 9d). Therefore, the inhibitory effect of the let-7i-3p mimic on the osteogenic differentiation of hASCs could be relieved by LEF1 overexpression under cyclic strain.

Compared to those in the group transfected with the let-7i-3p inhibitor and siR-Ctrl, the mRNA levels of the above markers were decreased 1.52 ± 0.26-fold (*p* = 0.026), 3.12 ± 1.55-fold (*p* = 0.002), 2.55 ± 0.25-fold (*p* = 0.038), 3.92 ± 0.72-fold (*p* = 0.018), and 2.25 ± 1.24-fold (*p* = 0.030), respectively, in hASCs cotransfected with the let-7i-3p inhibitor and siLEF1 (Fig. 9e); the protein expression levels of the above markers were also decreased 2.00 ± 0.42-fold (*p* = 0.002), 1.54 ± 0.17-fold (*p* = 0.013), 1.71 ± 0.19-fold (*p* = 0.003), 2.24 ± 0.28-fold (*p* = 0.001), and 1.55 ± 0.06-fold (*p* = 0.012), respectively (Fig. 9f). The immunofluorescence intensity of β-catenin in the hASC nucleus was also decreased in hASCs cotransfected with the let-7i-3p inhibitor and siLEF1 (Fig. 9g). Compared to those in the group transfected with the let-7i-3p inhibitor and siR-Ctrl, the protein expression levels of cytoplasmic and nuclear β-catenin were decreased 1.41 ± 0.19-fold (*p* = 0.036) and 2.13 ± 0.35-fold (*p* = 0.001), respectively, in hASCs cotransfected with the let-7i-3p inhibitor and siLEF1 (Fig. 9h).

Compared to those in the group transfected with the let-7i-3p mimic and EX-Ctrl, the mRNA expression levels of RUNX2, ALP, SPARC, and β-catenin were increased 2.42 ± 0.92-fold (*p* = 0.004), 4.27 ± 1.78-fold (*p* = 0.002), 3.06 ± 0.41-fold (*p* = 0.001), and 3.17 ± 1.09-fold (*p* = 0.008), the mRNA expression levels of LEF1 was decreased 1.47 ± 0.21-fold (*p* = 0.016), respectively, in hASCs cotransfected with the let-7i-3p mimic and EX-β-catenin (Fig. 9i); the protein expression levels of the above markers were also increased: RUNX2, ALP, SPARC, and β-catenin were increased 1.57 ± 0.06-fold (*p* = 0.011), 2.10 ± 0.03-fold (*p* = 0.001), 3.16 ± 1.01-fold (*p* = 0.002), and 1.50 ± 0.04-fold (*p* = 0.006); the protein expression levels of LEF1 were decreased 1.40 ± 0.13-fold (*p* = 0.023), respectively (Fig. 9j). The immunofluorescence intensity of β-catenin in the hASC nucleus was also increased in hASCs cotransfected with the let-7i-3p mimics and EX-β-catenin (Fig. 9k). Compared to those in the group transfected with the let-7i-3p mimic and EX-Ctrl, the protein expression levels of cytoplasmic and nuclear β-catenin were increased 1.35 ± 0.12-fold (*p* = 0.019) and 2.23 ± 0.27-fold (*p* = 0.008), respectively, in hASCs cotransfected with the let-7i-3p mimic and EX-β-catenin (Fig. 9l).

Compared to those in the group transfected with the let-7i-3p inhibitor and siR-Ctrl, the mRNA levels of RUNX2, ALP, SPARC, and β-catenin were decreased 1.73 ± 0.24-fold (*p* = 0.015), 2.63 ± 0.69-fold (*p* = 0.004), 2.36 ± 0.46-fold (*p* = 0.006), and 3.75 ± 0.21-fold (*p* = 0.001); the mRNA expression levels of LEF1 was increased 1.89 ± 0.74-fold (*p* = 0.009), respectively, in hASCs cotransfected with the let-7i-3p inhibitor and siβ-catenin (Fig. 9m); the protein expression levels of RUNX2, ALP, SPARC, and β-catenin were decreased 1.48 ± 0.15-fold (*p* = 0.007), 2.12 ± 0.14-fold (*p* = 0.006), 1.40 ± 0.01-fold (*p* = 0.020), and 1.51 ± 0.17-fold (*p* = 0.011); the protein expression levels of LEF1 were increased 1.53 ± 0.19-fold (*p* = 0.006), respectively (Fig. 9n). The immunofluorescence intensity of β-catenin in the hASC nucleus was also decreased in hASCs cotransfected with the let-7i-3p inhibitor and siβ-catenin (Fig. 9o). Compared to those in the group transfected with the let-7i-3p inhibitor and siR-Ctrl, the protein expression levels of cytoplasmic and nuclear β-catenin were decreased 1.35 ± 0.09-fold (*p* = 0.006) and 1.83 ± 0.04-fold (*p* = 0.004), respectively, in hASCs cotransfected with the let-7i-3p inhibitor and siβ-catenin (Fig. 9p).

Therefore, the potentiation of the let-7i-3p inhibitor on the osteogenic differentiation of hASCs could be weakened by LEF1 downregulation under cyclic strain.

## Discussion

Mechanical stimulation plays a vital role in bone formation and remolding and promotes angiogenesis. Bhatt et al. reported that a 3% cyclic strain increased the expression of OCN, OPN, and osteonectin in osteoblasts, and a 9% strain further enhanced cell proliferation [26]. A study on cell lines showed that the expression of RUNX2 and COL-1 in BMSCs increased with a 0–9% increase in tensile strain [27]. Some studies have shown that mechanical stimulation can promote the osteogenic differentiation and proliferation of bone MSCs and ASCs [27, 28]. Li et al. reported that mechanical strain could inhibit the expression of PPARγ to impede differentiation into adipocytes [29]. However, mechanical stimulation, as an independent factor, could promote the efficiency of constructing tissue-engineered bone.

The Wnt signaling pathway regulates the process of osteogenic differentiation for MSCs and is involved in intramembranous and endochondral ossification [30]. Specific genes for osteogenic differentiation, such as RUNX2, Dlx5, and Osterix, can be upregulated by the activated Wnt/β-catenin pathway, thereby promoting the osteogenic differentiation of BMSCs [31, 32]. β-catenin can promote the progression of MSCs from osteoblastic precursor cells into more mature osteoblasts and can also suppress the differentiation of MSCs into adipogenic and chondrogenic lineages [33, 34]. The Wnt/β-catenin pathway inhibits the expression of PPARγ and CCAAT/enhancer-binding protein α, the major adipogenic inducers, to inhibit adipogenic differentiation [35]. Additionally, mechanical stimulation can activate Wnt signaling pathways to promote the differentiation of stem cells into osteoblasts [36, 37]. Zhang et al. found that applying a static pressure of 100 kPa to periodontal ligament stem cells activates the Wnt/β-catenin pathway and regulates osteogenic differentiation [38]. Several studies have shown that tensile stress stimulation can also activate the Wnt/β-catenin and Wnt/Ca^2+^ pathways to promote the osteogenic differentiation of hASCs [39]. Evidence has shown that the Wnt signaling pathway regulates the osteogenic differentiation of MSCs [40]. In this study, we utilized cyclic strain to activate the Wnt/β-catenin pathway and thus induce the osteogenic differentiation of hASCs. There might be some differences in the protocol for using Wnt/β-catenin pathway activation to induce osteogenic differentiation compared to other methods, such as the conditioned medium, growth factors, and biomaterials. The mechanism by which the Wnt signaling pathway is activated by mechanical stimulation and the difference between using mechanical and chemical stimulation to activate this signaling pathway are still not understood.

LEF1 is an essential member of the effector protein family TCF/LEF that acts at the end of the Wnt signaling pathway. This protein forms a dimer with excessively aggregated β-catenin in the activated Wnt signaling pathway in the cytoplasm. Then, these complexes enter the nucleus to promote gene transcription and thus play regulatory roles in signaling. LEF1 plays a crucial regulatory role in the development of bone [41]. Hoeppner et al. reported that LEF1Δn binds β-catenin, stimulates LEF/TCF reporter activity, and promotes terminal osteoblast differentiation [42]. LEF1 transcription factors play essential roles in proper osteogenesis and bone volume loss in LEF1^+/−^ mice in the early years of life [43]. In the present study, we reduced the expression of LEF1 and significantly inhibited the osteogenic differentiation of hASCs. This result is generally consistent with the above findings.

Let-7i-3p, a type of microRNA (miRNA), is involved in many different types of diseases. Falzone et al. reported that abnormal let-7i-3p expression has the potential to predict recurrence in oral cancer patients [44]. Indersie et al. found that let-7i-3p expression was decreased in pediatric hepatoblastoma and inhibited hepatoblastoma cell growth and Wnt signaling activity in vitro partly through β-catenin downregulation [45]. In type 2 diabetes patients, the urinary extracellular vesicle miRNA signature had increased levels of let-7i-3p, which is a noninvasive early biomarker of diabetic nephropathy in patients with type 2 diabetes with the “Asian Indian phenotype” [46]. Gu et al. reported that let-7i-3p was significantly overexpressed in the serum of mandibular prognathism patients, indicating that let-7i-3p might be associated with bone development and growth [47]. Mandibular prognathism is characterized by bone overdevelopment and growth, which appears to be inconsistent with our findings. We found that overexpression of let-7i-3p could inhibit the osteogenic differentiation of hASCs. The reasons for this seemingly opposite result need to be studied further. Thus far, we are unaware of any study on the expression of let-7i-3p affecting bone formation, and this study is the first report on this issue.

Studies have been conducted to directly regulate the expression of LEF1 by miRNAs, mainly focusing on tumor diseases. miR-34a increased chemosensitivity in BIU87/ADR cells by targeted inhibition of the TCF1/LEF1 axis [48]. miR-22, a direct target of H19, binds to the 3′UTR of LEF1 to inhibit its expression and reverse the effect of H19 on nucleus pulposus cells, thereby inhibiting the Wnt/β-catenin pathway [49]. Overexpression of miR-708 inhibits the proliferation, invasion, migration, and epithelial-mesenchymal transition but also promotes the apoptosis of melanoma cells by targeting LEF1 through suppression of the Wnt signaling pathway [50]. The expression of miR-219-5p was shown to be decreased in colorectal cancer and to inhibit colorectal cancer metastasis and epithelial-mesenchymal transition by targeting LEF1 to inactivate the AKT and ERK pathways [51]. Suppressed miR-557 negatively regulates the expression of LEF1 to inhibit the proliferation of lung cancer cells [52].

Currently, some studies have confirmed that some miRNAs can affect the expression of LEF1 and the Wnt/β-catenin pathway in the process of osteogenesis. Overexpression of miR-539 increased the expression of β-catenin, LEF1, c-myc, cyclin D1, RUNX2, BGP, and BMP-2 in rat osteoblasts [53]. Overexpression of miR-10a inhibited the osteogenic differentiation of MC3T3-E1 cells by inactivating the Wnt signaling pathway; the expression of LEF1 was also decreased during this process [54]. However, reports of miRNAs directly regulating LEF1 and affecting osteogenesis have not been published. This study is the first to investigate the direct regulation of LEF1 expression by miRNAs and show that a miRNA affects the osteogenic differentiation of hASCs.

## Conclusion

**Fig. 10:**
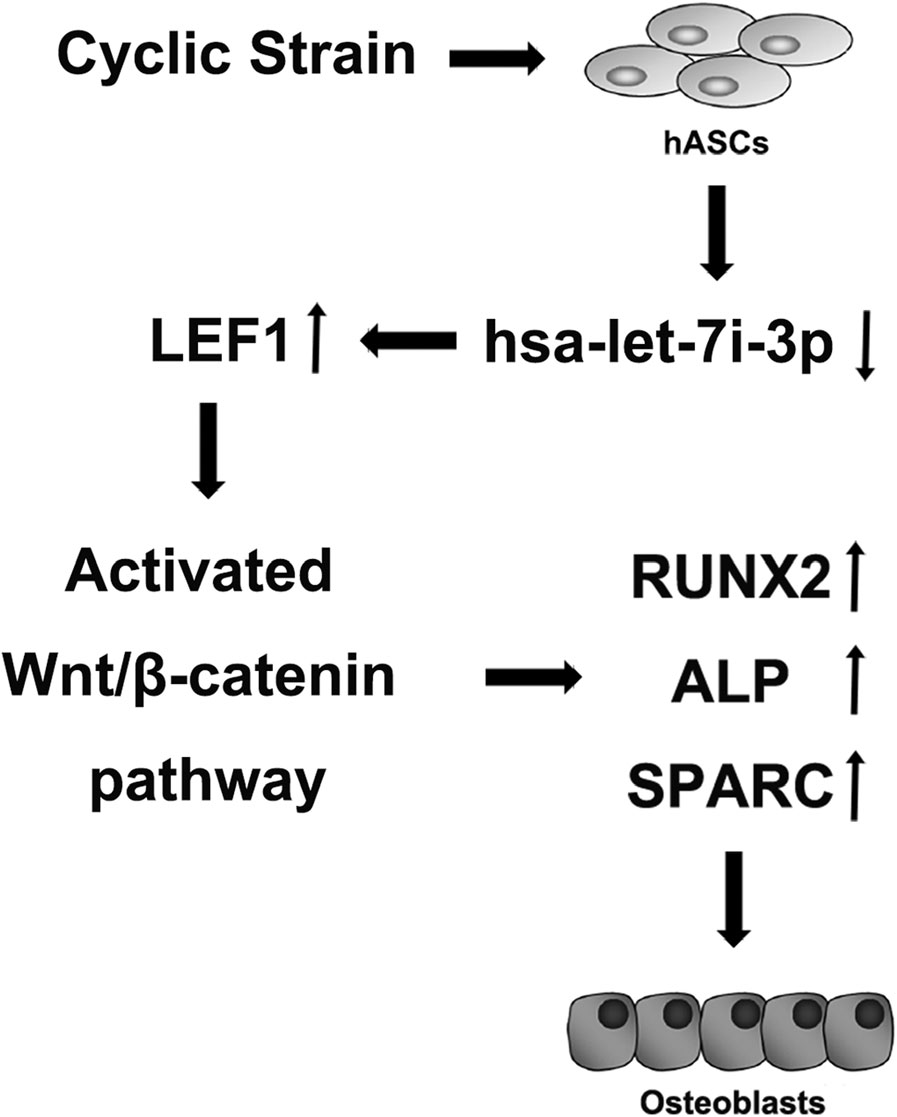
Let-7i-3p, as a negative regulator of the Wnt/β-catenin pathway by targeting LEF1, inhibits the osteogenic differentiation of hASCs under cyclic strain in vitro

To determine the role of let-7i-3p in the process of hASC osteogenic differentiation under cyclic strain, we designed serial experiments to verify its function. Inhibition of let-7i-3p and overexpression of LEF1 promoted the osteogenic differentiation of hASCs in vitro. Furthermore, gain- and loss-of-function experiments on let-7i-3p showed that let-7i-3p negatively regulates the Wnt/β-catenin pathway via LEF1, which inhibits the osteogenic differentiation of hASCs under cyclic strain in vitro (Fig. 10).

## Supplementary information

**Additional file 1.** The sequences mentioned in the study

**Additional file 2: Figure S1.** Microarray for miRNA changes under cyclic strain. There were 150 miRNAs decreased and 87 miRNAs increased significantly (>2-fold).

## Data Availability

The datasets generated and/or analyzed during the current study are not publicly available due but are available from the corresponding author upon reasonable request.

## Abbreviations

hASCs: Human adipose-derived stem cells
miRNAs: MicroRNAs
LEF1: Lymphoid enhancer factor 1
BMSCs: Bone marrow mesenchymal stem cells
PBS: Phosphate-buffered saline
α-MEM: Alpha-modified Eagle’s medium
FBS: Fetal bovine serum
CCK-8: Cell Counting Kit-8 assay
qPCR: Real-time quantitative PCR
SDS: Sodium dodecyl sulfate
EX-LEF1: LEF1 expression plasmid
siLEF1: LEF1 siRNA
EX-Ctrl: Negative control expression plasmid
siR-Ctrl: Negative control siRNA
miR-Ctrl: Mimic negative control of the let-7i-3p mimic
miR-Ctrl: Inhibitor negative control of the let-7i-3p inhibitor
wt-LEF1: LEF1 3′UTR-wild type
mu-LEF1: LEF1 3′UTR-mutant
ALP: Alkaline phosphatase
RUNX2: Runt-related transcription factor 2
SPARC: Secreted protein acidic and cysteine-rich
GAPDH: Glyceraldehyde-3-phosphate dehydrogenase
ANOVA: Analysis of variance
