# Supplementary material for: The osteogenic differentiation of human adipose-derived stem cells is regulated through the let-7i-3p/LEF1/β-catenin axis under cyclic strain
Source: Stem Cell Res Ther. 2019 Nov 21;10:339. doi: 10.1186/s13287-019-1470-z (PMC6873506; doi:10.1186/s13287-019-1470-z)

|  |
| --- |

**ORF Sequence Information for EX-LEF1**

| **Catalog No.:** EX-W0594-M02 | |  |
| --- | --- | --- |
| **Accession No.:**NM_016269 | **ORF Length:**1200 bp |  |
| **Whole Plasmid Size:**7023 bp | |  |
| **Description:**Homo sapiens lymphoid enhancer-binding factor 1 (LEF1), transcript variant 1, mRNA. | |  |
| **Vector:**pEZ-M02 | **Antibiotic:**Ampicillin |  |
| **Stable Selection Marker:**Neomycin | |  |
| **Suggested Sequencing Primers:** Forward: 5'-CAGCCTCCGGACTCTAGC-3' Reverse: 5'-TAATACGACTCACTATAGGG-3' | |  |
|  | | |

**ORF Sequence Information for EX-W0594-M02**

>EX-W0594-M02 ORF sequence

ATGCCCCAACTTTCCGGAGGAGGTGGCGGCGGCGGGGGGGACCCGGAACTCTGCGCCACGGACGAGATGATCCCCTTCAAGGACGAGGGCGATCCTCAGAAGGAAAAGATCTTCGCCGAGATCAGTCATCCCGAAGAGGAAGGCGATTTAGCTGACATCAAGTCTTCCTTGGTGAACGAGTCTGAAATCATCCCGGCCAGCAACGGACACGAGGTGGCCAGACAAGCACAAACCTCTCAGGAGCCCTACCACGACAAGGCCAGAGAACACCCCGATGACGGAAAGCATCCAGATGGAGGCCTCTACAACAAGGGACCCTCCTACTCGAGTTATTCCGGGTACATAATGATGCCAAATATGAATAACGACCCATACATGTCAAATGGATCTCTTTCTCCACCCATCCCGAGAACATCAAATAAAGTGCCCGTGGTGCAGCCATCCCATGCGGTCCATCCTCTCACCCCCCTCATCACTTACAGTGACGAGCACTTTTCTCCAGGATCACACCCGTCACACATCCCATCAGATGTCAACTCCAAACAAGGCATGTCCAGACATCCTCCAGCTCCTGATATCCCTACTTTTTATCCCTTGTCTCCGGGTGGTGTTGGACAGATCACCCCACCTCTTGGCTGGCAAGGTCAGCCTGTATATCCCATCACGGGTGGATTCAGGCAACCCTACCCATCCTCACTGTCAGTCGACACTTCCATGTCCAGGTTTTCCCATCATATGATTCCCGGTCCTCCTGGTCCCCACACAACTGGCATCCCTCATCCAGCTATTGTAACACCTCAGGTCAAACAGGAACATCCCCACACTGACAGTGACCTAATGCACGTGAAGCCTCAGCATGAACAGAGAAAGGAGCAGGAGCCAAAAAGACCTCACATTAAGAAGCCTCTGAATGCTTTTATGTTATACATGAAAGAAATGAGAGCGAATGTCGTTGCTGAGTGTACTCTAAAAGAAAGTGCAGCTATCAACCAGATTCTTGGCAGAAGGTGGCATGCCCTCTCCCGTGAAGAGCAGGCTAAATATTATGAATTAGCACGGAAAGAAAGACAGCTACATATGCAGCTTTATCCAGGCTGGTCTGCAAGAGACAATTATGGTAAGAAAAAGAAGAGGAAGAGAGAGAAACTACAGGAATCTGCATCAGGTACAGGTCCAAGAATGACAGCTGCCTACATCTAG

**Vector Information for EX-W0594-M02**


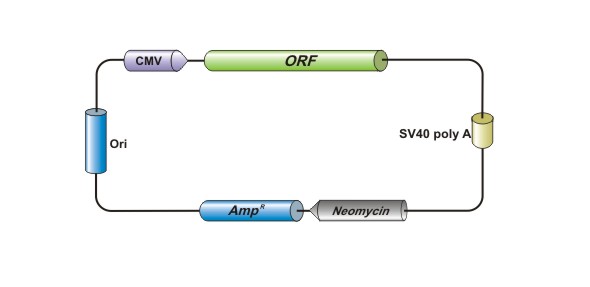


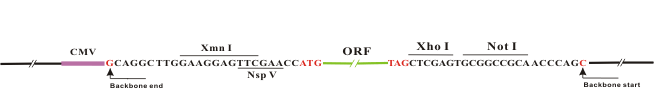


**ORF Sequence Information for EX-Ctrl**

|  |
| --- |

| **Catalog No.:** EX-NEG-M02 | |  |
| --- | --- | --- |
| **Description:**Negative control | |  |
| **Vector:**pReceiver-M02 | **Whole Plasmid Size:**5798 bp |  |
| **Stable Selection Marker:**Neomycin | **Antibiotic:**Ampicillin |  |
| **Suggested Sequencing Primers:** Forward: 5'-CAGCCTCCGGACTCTAGC-3' Reverse: 5'-TAATACGACTCACTATAGGG-3' | |  |
|  | | |

**Vector Information for EX-NEG-M02**


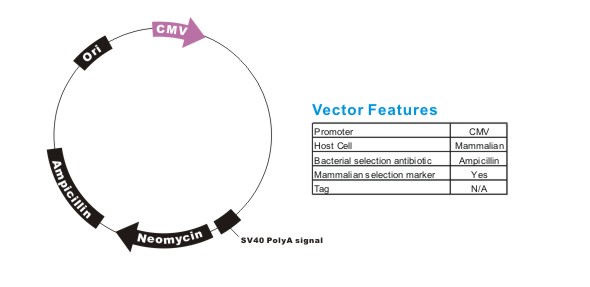

Supplement: Supplementary file 1 — Additional file 1. The sequences mentioned in the study [file 13287_2019_1470_MOESM1_ESM.docx]
