# Supplementary figures and images for: The osteogenic differentiation of human adipose-derived stem cells is regulated through the let-7i-3p/LEF1/β-catenin axis under cyclic strain
Source: Stem Cell Res Ther. 2019 Nov 21;10:339. doi: 10.1186/s13287-019-1470-z (PMC6873506; doi:10.1186/s13287-019-1470-z)

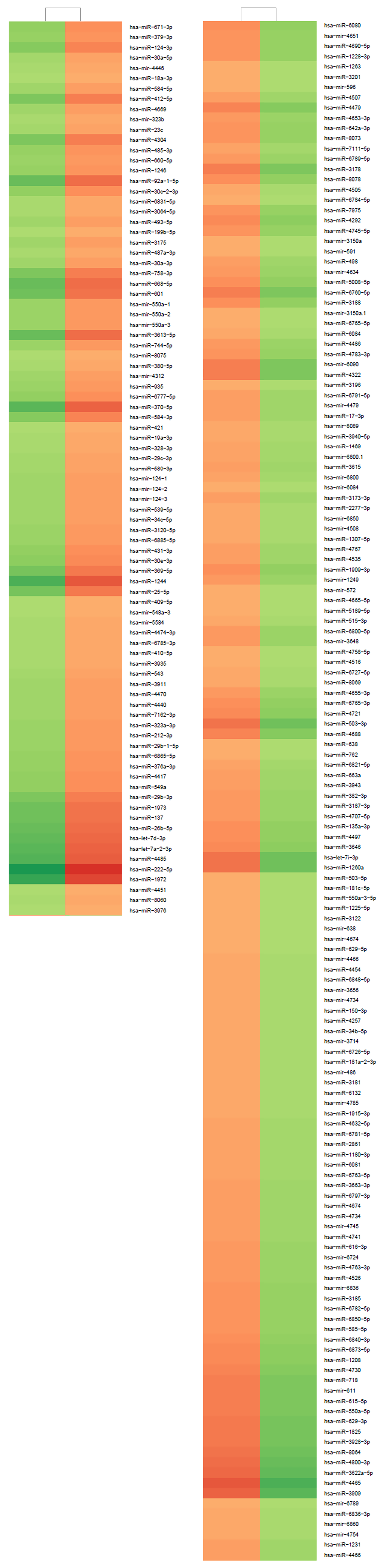

Supplement: Supplementary file 2 — Additional file 2: Figure S1. Microarray for miRNA changes under cyclic strain. There were 150 miRNAs decreased and 87 miRNAs increased significantly (>2-fold). [file 13287_2019_1470_MOESM2_ESM.tif]
